# Covalent Affibody‐Molecular Glue Drug Conjugate Nanoagent for Proximity‐Enabled Reactive Therapeutics

**DOI:** 10.1002/advs.202412273

**Published:** 2025-01-17

**Authors:** Wenhui Gao, Xiaoyuan Yang, Qingrong Li, Yingchun Liu, Wei Huang, Xuelin Xia, Deyue Yan

**Affiliations:** ^1^ School of Chemistry and Chemical Engineering Frontiers Science Center for Transformative Molecules Shanghai Jiao Tong University Shanghai 200240 China; ^2^ XIANGFU Laboratory, Jiaxing Zhejiang 314102 China

**Keywords:** affibody‐molecular glue drug conjugate, covalent nanoagent, proximity‐enabled reactive therapeutics, SuFEx reaction

## Abstract

Sulfur‐fluoride exchange (SuFEx) reaction is an emerging class of click chemistry reaction. Owing to its efficient reactivity under physiological conditions, SuFEx reaction is used to construct covalent protein drugs. Herein, a covalent affibody‐molecular glue drug conjugate nanoagent is reported, which can irreversibly bind with its target protein through proximity‐enabled SuFEx reaction. As a proof of concept, a latent bioreactive unnatural amino acid fluorosulfate‐L‐tyrosine (FSY) is first introduced at site 36 of the affibody with cysteine mutation (Z_HER2:342_‐Cys) to produce Z_HER2:342_‐36_FSY_‐Cys. Subsequently, Z_HER2:342_‐36_FSY_‐Cys is coupled with a molecular glue drug (CR8) to yield an amphiphilic conjugate of Z_HER2:342_‐36_FSY_‐CR8, which can self‐assemble into affibody–drug conjugate nanoagent (Z_HER2:342_‐36_FSY_‐CR8 ADCN) in PBS. When Z_HER2:342_‐36_FSY_‐CR8 ADCN specific binds to human epidermal growth factor receptor 2 (HER2) on cancer cells, the FSY36 of Z_HER2:342_ approaches to the His490 of HER2 and ultimately reacts with each other to form a covalent bond via SuFEx reaction. Such a covalent binding mode endows Z_HER2:342_‐36_FSY_‐CR8 ADCN with permanent binding ability to effectively increase the concentration of drugs in tumor. Eventually, the covalent Z_HER2:342_‐36_FSY_‐CR8 ADCN exhibits an outstanding tumor inhibition ratio of 90.03 ± 4.29% in HER2‐positive ovary tumor models, strikingly higher than that of the noncovalent one (64.25 ± 7.71%).

## Graphical abstract

The latent bioreactive unnatural amino acid fluorosulfate‐L‐tyrosine (FSY) at site 36 of affibody‐molecular glue drug conjugate (Z_HER2:342_‐36_FSY_‐CR8) comes into close proximity with the histidine at site 490 of HER2 (human epidermal growth factor receptor 2) and ultimately reacts with each other to form a covalent bond via proximity‐enabled sulfur‐fluoride exchange (SuFEx) reaction, as a result, covalent Z_HER2:342_‐36_FSY_‐CR8 irreversibly binds to HER2 on cancer cells.

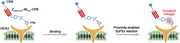

## 1 Introduction

Sulfur‐fluoride exchange (SuFEx) reaction is a novel class of click chemistry reactions developed in the laboratory of Karl Barry Sharpless, enabling efficient intermolecular linkage between sulfur (VI) fluorides and nucleophile.^[^ ^1^ ^]^ Sulfur (VI) fluorides exhibit the high stability against hydrolysis, thermolysis, and reduction as well as non‐reactivity toward free amino acids under physiological conditions. As the warhead of chemical probes, sulfonyl fluorine groups can be selectively activated by side chain groups of nucleophilic amino acids (such as cysteine, lysine, histidine, and tyrosine) near the binding interface of target protein to undergo SuFEx reactions and form robust covalent bonds.^[^ ^2^ ^]^ SuFEx reaction quickly becomes a powerful tool in the fields of chemical biology, drug discovery, and biotherapeutics on the base of its proximity‐enabled reactivity.

Utilizing the unique reactivity of SuFEx reaction, several sulfonyl fluorine containing protein drugs have been developed for cancer therapy.^[^ ^3^ ^]^ For example, Wang group successfully synthesized an unnatural amino acid fluorosulfate‐L‐tyrosine (FSY) with the latent chemical reactivity and remaining inert inside the protein and in vivo, and then introduced it into protein drugs by the genetic code expansion.^[^ ^4^ ^]^ When such a protein drug bound to its target, FSY was brought into close proximity to a natural residue of the target and reacted with it each other to form a covalent linkage between the drug and the target specifically.^[^ ^5^ ^]^ As a typical example, they engineered a programmed cell death protein‐1 (PD‐1) containing the FSY mutation. The resulting PD‐1(FSY) was able to covalently bind to its natural ligand PD‐L1 only both in vitro and in vivo, which resulted in a significantly potent antitumor response compared to that of the wild‐type noncovalent PD‐1. Its therapeutic efficacy was equivalent or superior to that of the commercially available anti‐PD‐L1 antibody atezolizumab in cancer immunotherapy.^[^ ^3a^ ^]^ Furthermore, Chen group introduced FSY into the nanobody to develop a covalent nanobody‐based PROTAC chimeras, which also exhibited enhanced degradation of membrane proteins and superior anticancer efficacy compared to that of non‐covalent counterparts.^[^ ^3d^ ^]^ In contrast to the reversible binding‐dissociation process of traditional protein/target interactions, such above covalent protein drugs can irreversibly and permanently bind to their targets, thereby effectively increasing drug accumulation at tumor sites, enhancing bioavailability, and achieving improved therapeutic outcomes.^[^ ^6^ ^]^ Therefore, the incorporation of FSY with covalent binding capability into drugs may be a powerful tool to enhance their pharmaceutical properties.

Affibodies, a class of non‐immunoglobulin single‐chain proteins comprising 58 amino acid residues, have received substantial interest in the past decade owing to its high picomolar level affinity and superior tissue‐penetrating ability.^[^ ^7^ ^]^ However, due to their small molecular size (6.5 kDa), affibodies are easily excreted by the kidneys, thus presenting inadequate pharmacokinetic properties that hinder their clinic application.^[^ ^8^ ^]^ Meanwhile, molecular glue drugs represent a class of novel and promising therapeutic molecules that utilize the ubiquitin‐proteasome pathway to degrade pathogenic proteins, and several candidates of them have approval for clinical application.^[^ ^9^ ^]^ However, further efforts are still required to address the undesired protein degradation at non‐target sites and enhance their therapeutic efficacy in vivo.^[^ ^10^ ^]^ In recent years, some affibodies were selected as targeting carrier to construct affibody‐drug conjugate nanoagent (ADCN) by the molecular self‐assembly strategy, and the obtained ADCNs exhibited extraordinary antitumor activity.^[^ ^11^ ^]^ However, the pharmacokinetic profile of ADCN is still limited and further improvement is needed.

The development of covalent modification strategy to drug molecules has been proved could effectively enhance the duration of drug action and improve the drug accumulation in action site, and minimize unintended binding reactions during circulation or in healthy tissues.^[^ ^3c^ ^]^ It is conceivable that if the affibody could deliver molecular glue drug in covalent mode, it would present a promising drug carrier with zero off‐rate and infinite affinity to its target, effectively improving the bioavailability of affibody and reducing the off‐target side effect of molecular glue drug.

**Scheme 1:**
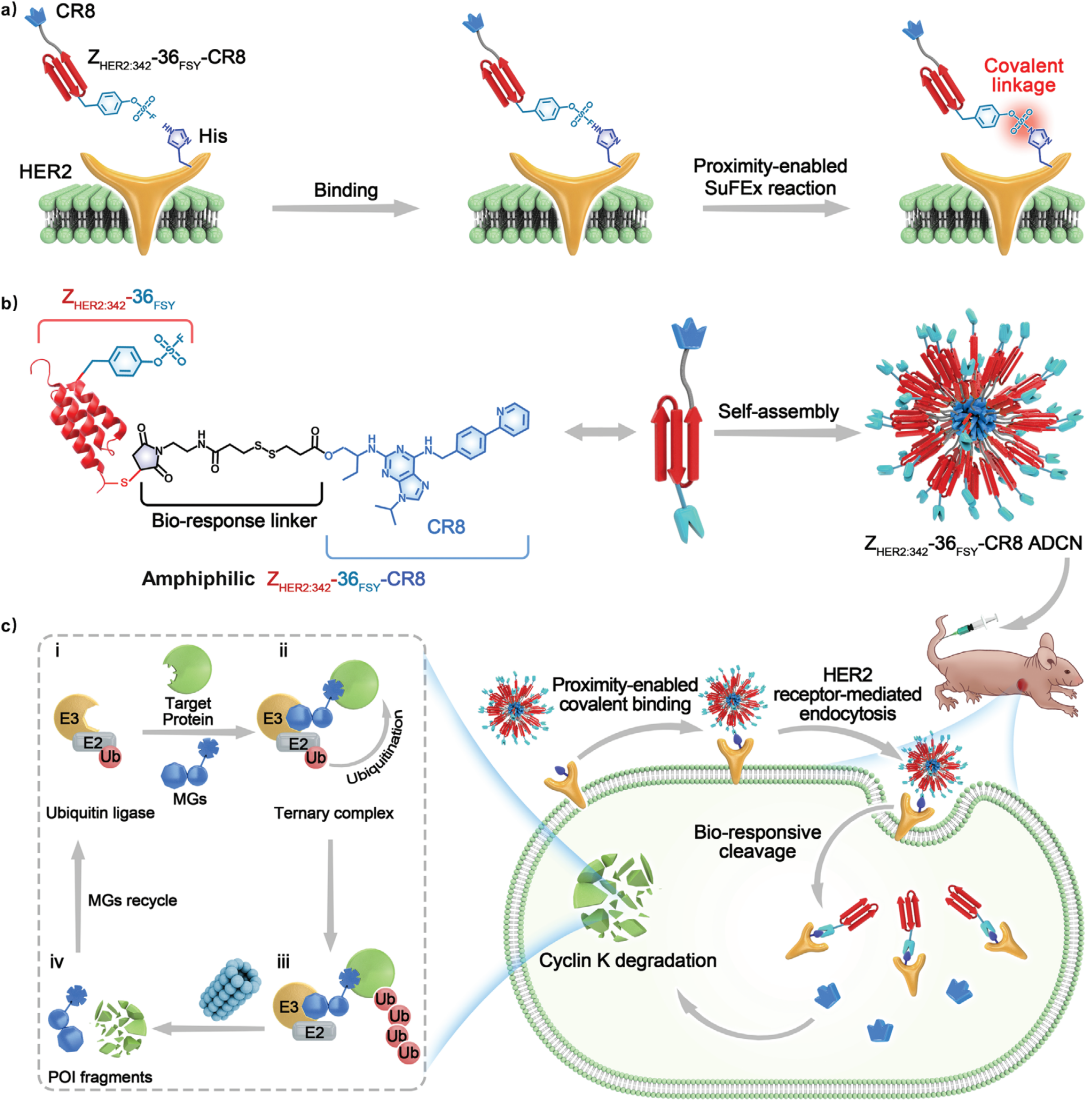
Schematic illustration of Z_HER2:342‐_36_FSY_‐CR8 ADCN for proximity‐enabled reactive therapeutics. a) Schematic representation of Z_HER2:342‐_36_FSY_‐CR8 binding covalently to HER2 via proximity‐enabled SuFEx reaction, whereas traditional protein‐target binding typically allows for dissociation. b) Schematic diagram of the structure and self‐assembly of the amphiphilic Z_HER2:342_‐36_FSY_‐CR8 conjugate, and its application in cancer therapy. Z_HER2:342‐_36_FSY_‐CR8 ADCN consisted of a hydrophobic core of CR8 and a hydrophilic shell of Z_HER2:342‐_36_FSY_. After internalized by cancer cells, CR8 was released through cleavage of disulfide bonds, inducing degradation of cyclin K. c) Mechanism of Z_HER2:342‐_36_FSY_‐CR8 ADCN mediated cyclin K degradation. i) Molecular glue (MG) CR8 recruits E3 ligase to form a ternary complex with the target protein and E3 ligase. ii, iii) The E3 ligase recruits a ubiquitin (Ub)‐tagged E2 ligase, facilitating the transfer of Ub to the POI and leading to its polyubiquitination. iv) The proteasome recognizes and degrades the polyubiquitinated POI, ultimately triggering apoptosis and death in cancer cells.

Herein, we construct a covalent affibody‐drug conjugate nanoagent by the self‐assembly of an amphiphilic conjugate made from FSY‐modified affibody and molecular glue drug for proximity‐enabled reactive therapeutics (**Scheme** **1**). First, FSY is incorporated at site 36 of Z_HER2:342_‐Cys to obtain Z_HER2:342_‐36_FSY_‐Cys via solid phase peptide synthesis. Subsequently, molecular glue drug CR8 is conjugated with Z_HER2:342_‐36_FSY_‐Cys through a bio‐responsive disulfide linker, the obtained affibody‐molecular glue drug conjugate can self‐assemble into nanoagent (Z_HER2:342_‐36_FSY_‐CR8 ADCN) in PBS. Upon binding of the Z_HER2:342_‐36_FSY_‐CR8 ADCN to its HER2 target, FSY36 getting close to the proximal His490 on HER2, resulting in a covalent linkage through proximity‐enabled SuFEx reaction. Different from the traditional reversible binding mode, Z_HER2:342_‐36_FSY_‐CR8 ADCN no longer dissociates once binding with HER2 in covalent mode, which significantly enhances the drug accumulation. After internalization into cancer cells, the CR8 payload of Z_HER2:342_‐36_FSY_‐CR8 ADCN is released via glutathione (GSH)‐triggered disulfide bond reduction. The free CR8 effectively catalyzes the degradation of cyclin K by the proteasome pathway, leading to potent cell apoptosis in vitro and in vivo.

## 2 Results and Discussions

### 2.1 Preparation and Characterization of Z_HER2:342_‐36_FSY_‐CR8 ADCN

Z_HER2:342_‐36_FSY_‐CR8 conjugate was synthesized according to the synthetic route in Scheme S1 (Supporting Information). In detail, CR8 was reacted with 3,3′‐dithiodipropionic acid via an esterification process to produce CR8‐ss‐COOH. Then, CR8‐ss‐COOH was further reacted with N‐(2‐aminoethyl) maleimide trifluoroacetic acid salt through an amidation reaction to yield CR8‐ss‐Mal. The chemical structure and molecular weight of CR8‐ss‐COOH and CR8‐ss‐Mal were verified by ^1^H NMR and MS results (Figures S1–S4, Supporting Information).

**Figure 1:**
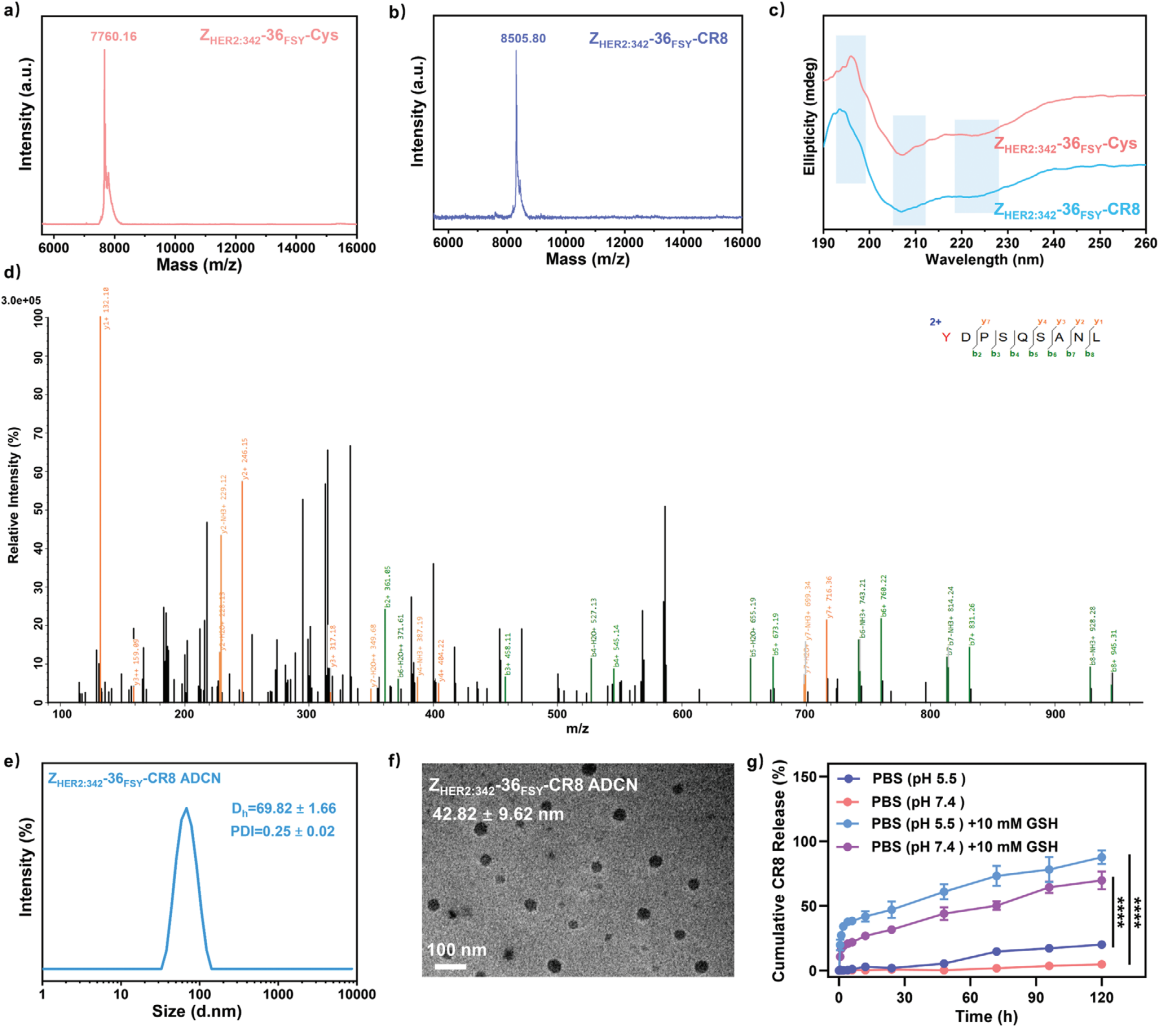
Characterization of Z_HER2:342_‐36_FSY_‐Cys, Z_HER2:342_‐36_FSY_‐CR8 conjugate and Z_HER2:342_‐36_FSY_‐CR8 ADCN. MALDI‐TOF‐MS spectra of a) Z_HER2:342_‐36_FSY_‐Cys and b) Z_HER2:342_‐36_FSY_‐CR8 conjugate. c) CD spectra of Z_HER2:342_‐36_FSY_‐Cys and Z_HER2:342_‐36_FSY_‐CR8 conjugate. d) Tandem mass spectrum of Z_HER2:342_‐36_FSY_‐Cys. Y represents FSY. e) Hydrodynamic diameter of Z_HER2:342_‐36_FSY_‐CR8 ADCN. f) TEM image of Z_HER2:342_‐36_FSY_‐CR8 ADCN. Scale bar = 100 nm. g) In vitro pH/GSH responsive CR8 release of Z_HER2:342_‐36_FSY_‐CR8 ADCN.

Z_HER2:342_‐36_FSY_‐Cys was synthesized via solid phase synthesis with the following amino acid sequence (HHHHHHVDNKFNKEMRNAYWEIALLPNLNNQQKRAFIRSLYY_FSY_DPSQSANLLAEAKKLNDAQAPKC), in which FSY was introduced at site 36 of Z_HER2:342_‐Cys. The resulting Z_HER2:342_‐36_FSY_‐Cys was analyzed by matrix‐assisted laser desorption/ionization time of flight mass spectrometry (MALDI‐TOF‐MS) to exhibit a single peak at 7760.16 (**Figure** **1**a), which was consistent with its theoretical molecular weight of 7761.58. Then Z_HER2:342_‐36_FSY_‐Cys was further identified with tandem MS, and a series of b and y ions confirmed that FSY was successfully incorporated at site 36 of Z_HER2:342_‐Cys (Figure 1d). Finally, Z_HER2:342_‐36_FSY_‐Cys was coupled with CR8‐ss‐Mal effectively by the thiol‐ene click reaction to produce Z_HER2:342_‐36_FSY_‐CR8 conjugate. As shown in Figure 1b, a single peak was observed at 8505.80, which was very close to the theoretical molecular weight of Z_HER2:342_‐36_FSY_‐CR8 conjugate (8507.50). Meanwhile, the secondary structure of Z_HER2:342_‐36_FSY_‐Cys and Z_HER2:342_‐36_FSY_‐CR8 conjugate was also confirmed by circular dichroism (CD) spectroscopy. As shown in Figure 1c, a distinct positive peak at approximately 195 nm and two negative peaks at 205 and 225 nm were observed in the CD spectra of both, which verified that α‐helical structure was still dominating in these Z_HER2:342_ derivatives to assure their high HER2‐binding affinity.^[^ ^12^ ^]^

Benefiting from its amphiphilic structure, Z_HER2:342_‐36_FSY_‐CR8 conjugate can self‐assemble into nanoagent (Z_HER2:342_‐36_FSY_‐CR8 ADCN) in PBS. The hydrodynamic diameter of Z_HER2:342_‐36_FSY_‐CR8 ADCN was 69.82 ± 1.66 nm with a narrow particle size distribution (PDI = 0.25 ± 0.02) measured by dynamic light scattering (DLS) (Figure 1e). The transmission electron microscopy (TEM) image of Z_HER2:342_‐36_FSY_‐CR8 ADCN revealed they were uniform spherical nanoparticles with an average diameter of 42.82 ± 9.62 nm (Figure 1f). Herein, the average diameter by TEM is slightly smaller than that by DLS, which is ascribed to the fact that the nanoparticles are in a dry shrinkage state in the TEM measurement, but they are in hydration expansion state in the DLS measurement. Moreover, the critical micellar concentration (CMC) of Z_HER2:342_‐36_FSY_‐CR8 conjugate was as low as 15.55 µg mL^−1^, further confirmed its strong self‐assembly ability in PBS (Figure S5, Supporting Information). In addition, the in vitro CR8 release of Z_HER2:342_‐36_FSY_‐CR8 ADCN was also investigated under various pH and GSH. As shown in Figure 1g, only 4.81 ± 0.23% of CR8 was released from Z_HER2:342_‐36_FSY_‐CR8 ADCN in PBS (pH = 7.4) within 120 h, suggesting it was quite stable under the physiological condition. As pH = 5.5, the accumulative release amount of CR8 from Z_HER2:342_‐36_FSY_‐CR8 ADCN was up to 20.10 ± 1.43% within 120 h. While pH = 5.5 and GSH = 10 mM, the accumulative release amount of CR8 from Z_HER2:342_‐36_FSY_‐CR8 ADCN was significantly enhanced to 87.55 ± 4.27% within 120 h. All above results verified that Z_HER2:342_‐36_FSY_‐CR8 ADCN possessed pH/GSH responsive release behavior and could rapid released CR8 under the acidic microenvironment and high level of GSH in tumor cells.

### 2.2 Z_HER2:342_‐36_FSY_‐CR8 ADCN Irreversibly Binding to HER2

**Figure 2:**
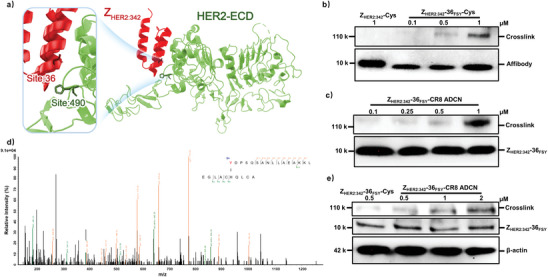
Characterization of Z_HER2:342_‐36_FSY_‐Cys and Z_HER2:342_‐36_FSY_‐CR8 ADCN irreversibly binding to HER2. a) Crystal structure schematic diagram of Z_HER2:342_/HER2 complex (PDB: 3MZW). b) Western blotting analysis of Z_HER2:342_‐Cys or Z_HER2:342_‐36_FSY_‐Cys binding to HER2‐ECD. c) Western blotting analysis of Z_HER2:342_‐36_FSY_‐CR8 ADCN binding to HER2‐ECD. d) Tandem mass spectrum of the product of Z_HER2:342_‐36_FSY_‐CR8 conjugate binding to HER2‐ECD. Y represents FSY. e) Western blotting analysis of Z_HER2:342_‐36_FSY_‐Cys and Z_HER2:342_‐36_FSY_‐CR8 ADCN binding to HER2 on SKOV‐3 cells surface.

According to previous reports, FSY can react with His to form a covalent linkage through a proximity‐enabled SuFEx reaction.^[^ ^3^, ^4^ ^]^ To verify the cross‐linking occurred between FSY36 on Z_HER2:342_‐36_FSY_‐CR8 ADCN and His490 on HER2 by the same reaction (**Figure** **2**a), western blotting and tandem MS analysis were performed. As shown in Figure 2b, a new band was observed at 110 kDa corresponding to the Z_HER2:342_‐36_FSY_‐Cys/HER2 complex, while not detected for the noncovalent Z_HER2:342_‐Cys/HER2 complex. In addition, Z_HER2:342_‐36_FSY_‐CR8 ADCN was able to covalently conjugate with HER2 in a dose‐dependent manner (Figure 2c). The cross‐linked Z_HER2:342_‐36_FSY_‐CR8 ADCN/HER2 complex was trypsin digested and then analyzed by tandem MS. As shown in Figure 2d, a series of b and y fragment ions clearly indicated that FSY36 in the affibody cross‐linked precisely with His490 in HER2. The above results collectively confirmed the extracellular covalent binding ability of Z_HER2:342_‐36_FSY_‐CR8 ADCN.

Furthermore, the intracellular covalent cross‐linking ability of Z_HER2:342_‐36_FSY_‐CR8 ADCN was also confirmed through western blotting analysis. SKOV‐3 cells (human ovarian adenocarcinoma cells) with the high HER2 expression level were treated with Z_HER2:342_‐36_FSY_‐Cys and Z_HER2:342_‐36_FSY_‐CR8 ADCN at 37 °C for 5 h, respectively. Then the cell lysates were immunoblotted under denatured conditions. As shown in Figure 2e, both Z_HER2:342_‐36_FSY_‐Cys and Z_HER2:342_‐36_FSY_‐CR8 ADCN irreversibly cross‐linked with the endogenous HER2 receptor on SKOV‐3 cells. This verified that Z_HER2:342_‐36_FSY_‐CR8 ADCN could irreversibly bind to HER2 via proximity‐enabled SuFEx reaction in cells.

### 2.3 The Increased Intracellular Uptake of Z_HER2:342_‐36_FSY_‐CR8 ADCN

**Figure 3:**
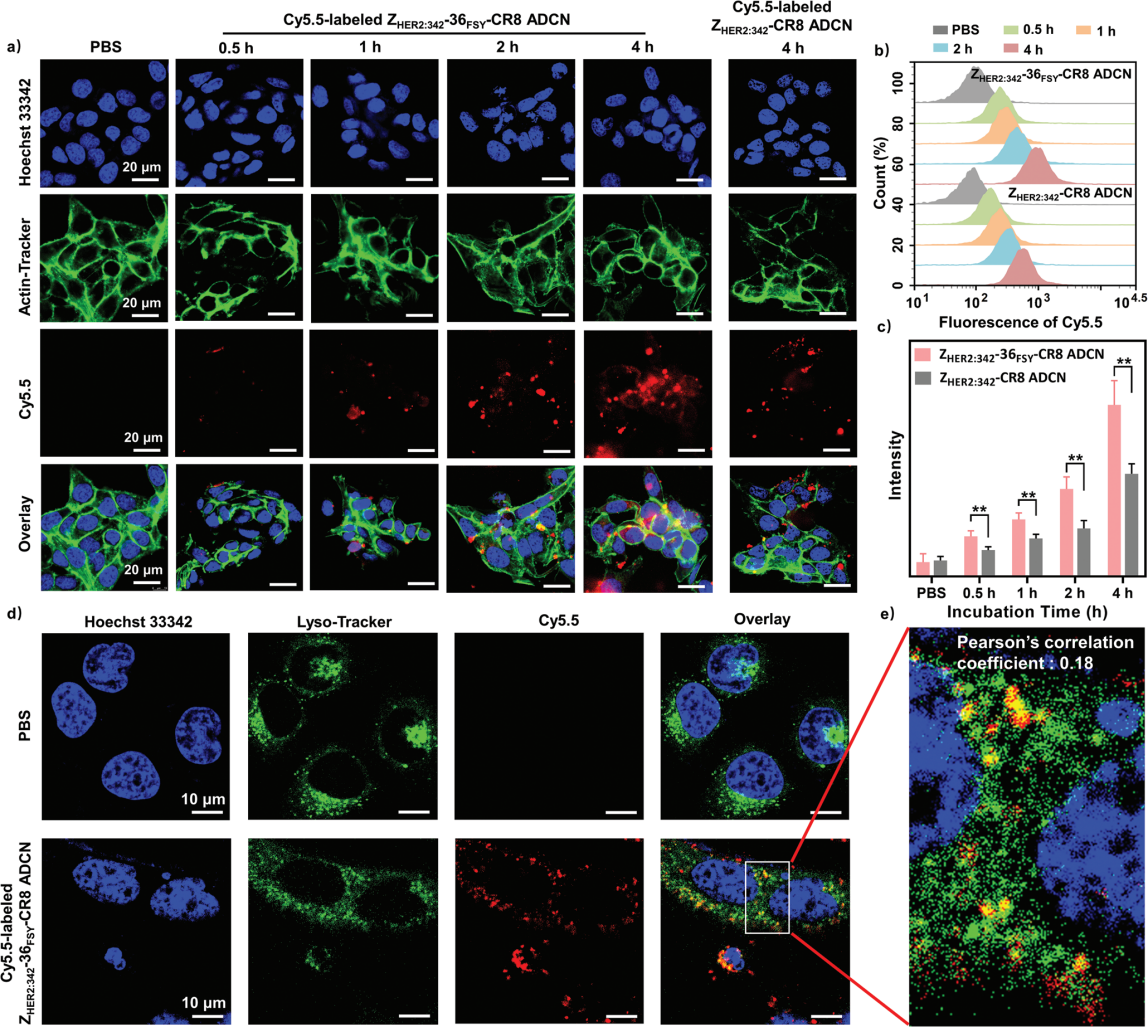
The increased intracellular uptake of Z_HER2:342_‐36_FSY_‐CR8 ADCN. a) CLSM images, b) flow cytometry image, and c) flow quantitative data of SKOV‐3 cells incubated with Cy5.5‐labeled Z_HER2:342_‐36_FSY_‐CR8 ADCN or Cy5.5‐labeled Z_HER2:342_‐CR8 ADCN (10 µg mL^−1^ of Cy5.5). Cy5.5 fluorescence (red), actin‐tracker (green), and Hoechst 33342 (blue). Scale bar = 20 µm. CLSM images d) and magnified zoom e) of lysosome location of Cy5.5‐labeled Z_HER2:342_‐36_FSY_‐CR8 ADCN (10 µg mL^−1^ of Cy5.5) after 12 h incubation in SKOV‐3 cells. Cy5.5 fluorescence (red), lyso‐tracker green (detect lysosome), and Hoechst 33342 (blue). Scale bar = 10 µm. Data are presented as mean ± SD (*n* = 3). ** *p* < 0.01, *t*‐test.

The introduction of SuFEx warheads into drug molecule can significantly enhance its intracellular accumulation.^[^ ^3c^ ^]^ Firstly, noncovalent Z_HER2:342_‐CR8 ADCN was constructed through the conjugation of original Z_HER2:342_‐Cys with CR8 as a negative control. The MALDI‐TOF‐MS and CD results confirmed the successful preparation of Z_HER2:342_‐CR8 conjugate (Figures S6 and S7, Supporting Information). The self‐assembly behavior of Z_HER2:342_‐CR8 conjugate to form Z_HER2:342_‐CR8 ADCN was similar to that of Z_HER2:342_‐36_FSY_‐CR8 conjugate (Figures S8 and S9, Supporting Information). Subsequently, Cy5.5‐labeled Z_HER2:342_‐36_FSY_‐CR8 ADCN was used to visualize the cellular uptake efficiency of them after the introduction of covalent binding ability. Both SKOV‐3 and ECa‐109 cells (human esophageal cancer cells) with high expression levels of HER2 and cyclin K were chosen to investigate the internalization process of Z_HER2:342_‐36_FSY_‐CR8 ADCN by confocal laser scanning microscopy (CLSM) and flow cytometry. As shown in **Figure** **3**a, a notable uptake of Z_HER2:342_‐36_FSY_‐CR8 ADCN was observed after 0.5 h of incubation, and the uptake efficiency gradually increased within 4 h. Compared with Z_HER2:342_‐36_FSY_‐CR8 ADCN, noncovalent Z_HER2:342_‐CR8 ADCN exhibited a lower uptake efficiency within 4 h incubation. Quantitative analysis of flow cytometry also demonstrated a higher time‐dependent uptake trend for Z_HER2:342_‐36_FSY_‐CR8 ADCN compared to that for noncovalent Z_HER2:342_‐CR8 ADCN (Figure 3b,c). In addition, ECa‐109 cells exhibited the similar cellular uptake behavior like SKOV‐3 cells (Figure S10, Supporting Information). These data demonstrated that Z_HER2:342_‐36_FSY_‐CR8 ADCN exhibited an enhanced cellular uptake ability.

The cellular internalization mechanism of Z_HER2:342_‐36_FSY_‐CR8 ADCN was further investigated by competitive inhibition assays. SKOV‐3 cells were first pre‐incubated with free Z_HER2:342_‐Cys for 1 h, and then they were co‐incubated with Cy5.5‐labeled Z_HER2:342_‐36_FSY_‐CR8 ADCN for another 4 h. Flow cytometry results in Figure S11 (Supporting Information) clearly indicated that pre‐incubation with free Z_HER2:342_‐Cys blocked the internalization of Z_HER2:342_‐36_FSY_‐CR8 ADCN by SKOV‐3 cells, confirming that Z_HER2:342_‐36_FSY_‐CR8 ADCN was internalized via HER2‐mediated endocytosis.

The lysosome escape capacity is essential for nanoagent to achieve their efficacy.^[^ ^11c^ ^]^ Here the intracellular co‐localization assay was performed on SKOV‐3 cells using Cy5.5‐labeled Z_HER2:342_‐36_FSY_‐CR8 ADCN, meanwhile lysosomes were stained with Lyso‐Tracker green. The CLSM images in Figure 3d demonstrated the minimal fluorescence overlap between Cy5.5 and Lyso‐Tracker green. Furthermore, the zoom‐in analysis in Figure 3e demonstrated the Pearson correlation coefficient was 0.18, which confirmed absence of significant fluorescence overlap. These results indicated that Z_HER2:342_‐36_FSY_‐CR8 ADCN could effectively escape from endosomes after cellular internalization.

### 2.4 The Degradation of Cyclin K Induced Anti‐Proliferation Activity

**Figure 4:**
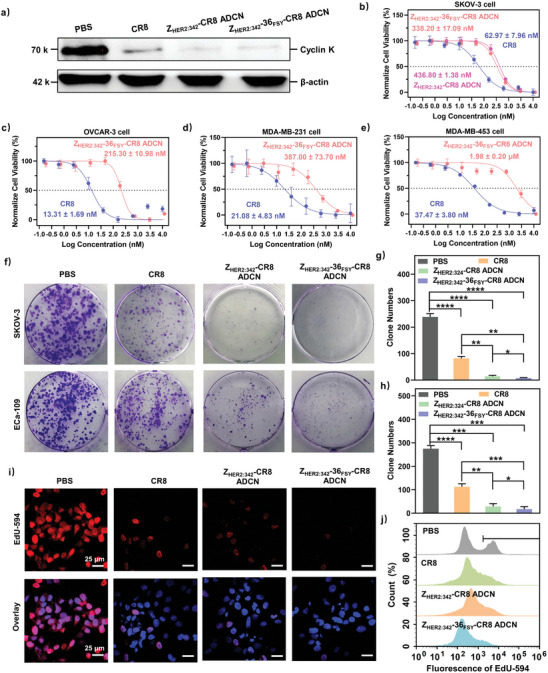
The degradation of cyclin K induced anti‐proliferation activity. a) The ability to degrade cyclin K in 24 h of CR8, Z_HER2:342_‐CR8 ADCN, and Z_HER2:342_‐36_FSY_‐CR8 ADCN determined by western blotting. b) Normalize cell viability of SKOV‐3 cells incubated with CR8, Z_HER2:342_‐CR8 ADCN or Z_HER2:342_‐36_FSY_‐CR8 ADCN for 48 h determined by CCK‐8 assay. Normalize cell viability of c) OVCAR‐3, d) MDA‐MB‐231, and e) MDA‐MB‐453 cells incubated with CR8 or Z_HER2:342_‐36_FSY_‐CR8 ADCN for 48 h determined by CCK‐8 assay. f) Colony formation images and g) colony formation numbers of SKOV‐3 and h) ECa‐109 cells treated with PBS, CR8, Z_HER2:342_‐CR8 ADCN, or Z_HER2:342_‐36_FSY_‐CR8 ADCN, respectively, at a dose of 100 nM. i) CLSM images, and j) flow cytometry analysis of EdU‐594 assays in SKOV‐3 cells treated with PBS, CR8, Z_HER2:342_‐CR8 ADCN, or Z_HER2:342_‐36_FSY_‐CR8 ADCN, respectively, at a dose of 500 nM. Azide 594 fluorescence (red), and Hoechst 33342 (blue). Scale bar = 25 µm. Data are presented as mean ± SD (*n* = 3). *p < 0.1, **p < 0.01, *** *p* < 0.001, and **** *p* < 0.0001, *t*‐test.

The ability of Z_HER2:342_‐36_FSY_‐CR8 ADCN to degrade cyclin K was further evaluated by western blotting analysis. SKOV‐3 cells were treated with PBS, CR8, Z_HER2:342_‐CR8 ADCN or Z_HER2:342_‐36_FSY_‐CR8 ADCN to assess their ability to degrade cyclin K. As shown in **Figure** **4**a, both Z_HER2:342_‐36_FSY_‐CR8 ADCN and Z_HER2:342_‐CR8 ADCN groups demonstrated a higher efficiency to degrade cyclin K compared to that of CR8 group, which was attributed to the efficient internalization of nanoagents by cells via HER2‐mediated endocytosis.

Afterwards, cell counting Kit‐8 (CCK‐8) assay was conducted to assess the cytotoxicity of Z_HER2:342_‐36_FSY_‐CR8 ADCN and Z_HER2:342_‐CR8 ADCN towards SKOV‐3 cells. As depicted in Figure 4b, the IC_50_ value of Z_HER2:342_‐36_FSY_‐CR8 ADCN against SKOV‐3 cells was 338.20 ± 17.09 nM, which was obviously lower than that of Z_HER2:342_‐CR8 ADCN (436.80 ± 1.38 nM). The enhanced cytotoxicity of Z_HER2:342_‐36_FSY_‐CR8 ADCN was attributed to its covalent binding capability to the target.

Generally, the cytotoxicity of affibody‐drug conjugate is associated with the expression levels of receptors in cancer cells.^[^ ^13^ ^]^ Thus, Z_HER2:342_‐36_FSY_‐CR8 ADCN may demonstrate the diverse cytotoxic to cells with various HER2 expression levels. Here we selected HER2‐positive expression cell lines (SKOV‐3 and ECa‐109 cells), HER2 low‐expression level lines (OVCAR‐3, human ovarian cancer cells; MDA‐MB‐231, human breast cancer cells), and HER2‐negative expression cell line (MDA‐MB‐453, human breast cancer cells) to assess the anti‐proliferative activity of CR8 and Z_HER2:342_‐36_FSY_‐CR8 ADCN by CCK‐8 assay. As depicted in Figure 4b and Figure S12 (Supporting Information), Z_HER2:342_‐36_FSY_‐CR8 ADCN demonstrated the significant cytotoxicity against SKOV‐3 and ECa‐109 cells, with IC_50_ values of 338.20 ± 17.09 nM and 160.33 ± 14.29 nM, respectively. In comparison to those of CR8 (62.97 ± 7.96 nM and 14.86 ± 1.87 nM), the elevation of IC_50_ values may be attributed to the delayed activation of bio‐responsive Z_HER2:342_‐36_FSY_‐CR8 ADCN.

Furthermore, the IC_50_ values of CR8 in OVCAR‐3, MDA‐MB‐231, and MDA‐MB‐453 cells were 13.31 ± 1.69 nM, 21.08 ± 4.83 nM, and 37.47 ± 3.80 nM, respectively (Figure 4c–e), which were approximate to those of HER2 high expression cancer cell lines mentioned above. This indicated that CR8 has no specific cytotoxicity to cancer cells. However, the IC_50_ values of Z_HER2:342_‐36_FSY_‐CR8 ADCN exhibited a significant increase compared to that of CR8, such as 16.18‐fold increase in OVCAR‐3 (215.30 ± 10.98 nM), 18.36‐fold increase in MDA‐MB‐231 (387.00 ± 73.70 nM), and 52.84‐fold increase in MDA‐MB‐453 (1.98 ± 0.20 µM) cells. The above results demonstrated that Z_HER2:342_‐36_FSY_‐CR8 ADCN exhibited the enhanced cytotoxicity towards cancer cells with the elevated HER2 expression, which was ascribed to the outstanding targeting ability of Z_HER2:342_‐36_FSY_.

In addition, anti‐proliferative effects of Z_HER2:342_‐36_FSY_‐CR8 ADCN were further investigated in SKOV‐3 and ECa‐109 cells by colony formation assay. As shown in Figure 4f, Z_HER2:342_‐36_FSY_‐CR8 ADCN exhibited the significantly higher inhibition of clonogenicity in both SKOV‐3 and ECa‐109 cells compared to those of CR8 and Z_HER2:342_‐CR8 ADCN. Meanwhile, the quantitative results in Figure 4g,h indicated that Z_HER2:342_‐36_FSY_‐CR8 ADCN led to a significantly lower clone numbers (7.67 ± 1.25% and 17.00 ± 8.64%), compared with those of Z_HER2:342_‐CR8 ADCN (14.33 ± 2.87% and 29.00 ± 9.42%), CR8 (82.00 ± 5.17% and 112.67 ± 10.20%), and PBS (238.67 ± 9.98% and 274.67 ± 10.66%). These results confirmed the excellent anti‐proliferative activity of Z_HER2:342_‐36_FSY_‐CR8 ADCN.

Subsequently, EdU assay was also conducted using Azide 594 dye to assess the anti‐proliferative activity of Z_HER2:342_‐36_FSY_‐CR8 ADCN. As shown in Figure 4i, the cells treated with PBS exhibited the strongest red fluorescence, indicating active cell proliferation. The cells treated with CR8 or Z_HER2:342_‐CR8 ADCN exhibited a relatively weak red fluorescence, indicating inactive cell proliferation. On the contrary, the cells treated with Z_HER2:342_‐36_FSY_‐CR8 ADCN only displayed ignorable red fluorescence, indicating the cell proliferation was completely inhibited. Moreover, quantitative analysis of flow cytometry using Azide 594 channel was employed to determine the proliferative status of cells in above groups. In Figure 4j, PBS group exhibited a negative peak (left, representing non‐proliferating cells) and a positive peak (right, representing proliferating cells) of 594 red fluorescence. For CR8, Z_HER2:342_‐CR8 ADCN, and Z_HER2:342_‐36_FSY_‐CR8 ADCN groups, the positive peak was reduced successively, indicating antiproliferative activity was enhanced gradually. Specifically, the 594 red fluorescence positive rate of PBS group was 34.86 ± 2.74%, but for other groups, it was gradually decreased to 19.75 ± 5.80% (CR8 group), 11.46 ± 1.02% (Z_HER2:342_‐CR8 ADCN group) and 5.88 ± 1.41% (Z_HER2:342_‐36_FSY_‐CR8 ADCN group) (Figure S13, Supporting Information). These results confirmed the significant anti‐proliferative activity of Z_HER2:342_‐36_FSY_‐CR8 ADCN.

The migratory capability of SKOV‐3 cells treated with various treatments was evaluated by cell scratch assay (Figure S14, Supporting Information). After 24 h of incubation, the cell migration rate of Z_HER2:342_‐36_FSY_‐CR8 ADCN group was 5.18 ± 1.98%, significantly lower than that of PBS (98.83 ± 1.12%), CR8 (50.84 ± 7.86%), and Z_HER2:342_‐CR8 ADCN (28.88 ± 3.62%) groups. This confirmed Z_HER2:342_‐36_FSY_‐CR8 ADCN could effectively inhibit the migration of HER2‐overexpressing cancer cells.

### 2.5 The Degradation of Cyclin K Induced Cell Apoptosis

**Figure 5:**
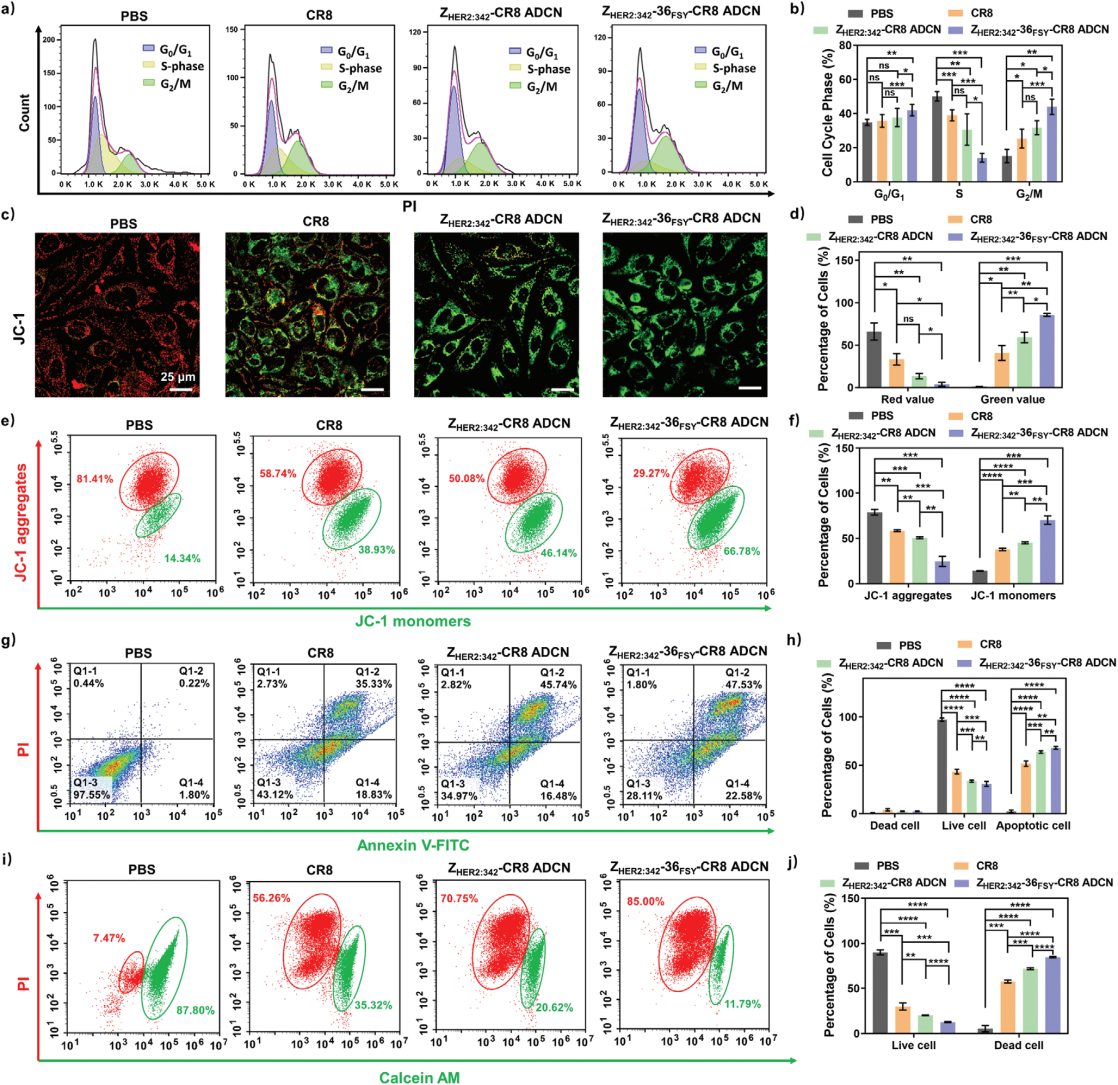
The degradation of cyclin K induced cell apoptosis. a) Cell cycle analysis and b) the corresponding quantitative data of SKOV‐3 cells treated with PBS, CR8, Z_HER2:342_‐CR8 ADCN, or Z_HER2:342_‐36_FSY_‐CR8 ADCN, respectively, at a dose of 200 nM. c) The CLSM images, e) flow cytometry images, and d,f) the corresponding quantitative data of JC‐1 staining assay for SKOV‐3 cells treated with PBS, CR8, Z_HER2:342_‐CR8 ADCN, or Z_HER2:342_‐36_FSY_‐CR8 ADCN, respectively, at a dose of 500 nM. JC‐1 aggregates (red), and JC‐1 monomers (green). Scale bar = 25 µm. g) Flow cytometry images and h) the corresponding quantitative data of Annexin V‐FITC/PI staining assay for SKOV‐3 cells treated with PBS, CR8, Z_HER2:342_‐CR8 ADCN, or Z_HER2:342_‐36_FSY_‐CR8 ADCN, respectively, at a dose of 500 nM. i) Flow cytometry images and j) the corresponding quantitative data of Calcein/PI staining assay for SKOV‐3 cells treated with PBS, CR8, Z_HER2:342_‐CR8 ADCN, or Z_HER2:342_‐36_FSY_‐CR8 ADCN, respectively, at a dose of 500 nM. Data are presented as mean ± SD (*n* = 3). *p < 0.1, **p < 0.01, ***p < 0.001, and ****p < 0.0001, ns: no significant, *t*‐test.

The cell cycle transcription is related to the activity of cyclins and cyclin‐dependent kinases.^[^ ^14^ ^]^ Here cell cycle distribution assay was performed to evaluate the effects of the degradation of cyclin K on cell cycle transcription. As shown in **Figure** **5**a,b, a significantly decreased proportion of DNA in the S phase was observed (14.08 ± 2.19%) for Z_HER2:342_‐36_FSY_‐CR8 ADCN group compared with those for PBS (50.10 ± 2.45%), CR8 (38.95 ± 2.82%), and Z_HER2:342_‐CR8 ADCN (30.58 ± 7.94%) groups. Meanwhile, Z_HER2:342_‐36_FSY_‐CR8 ADCN group showed a higher percentage of DNA in the G_2_/M phase (43.95 ± 3.84%) compared to those of other groups (PBS: 15.08 ± 3.38%, CR8: 25.33 ± 4.80%, and Z_HER2:342_‐CR8 ADCN: 31.73 ± 3.55%). These results indicated that Z_HER2:342_‐36_FSY_‐CR8 ADCN could block cell cycle in G_2_/M transition phase, which was consistent with the result in previous study.^[^ ^15^ ^]^

Early apoptosis assay was conducted by using JC‐1 as a fluorescent marker. The JC‐1 fluorescence shift from red to green was served as an indicator for detecting early apoptosis. As shown in Figure 5c,d, PBS group exhibited the stronger red fluorescence and weaker green fluorescence evidently. But for CR8, Z_HER2:342_‐CR8 ADCN, and Z_HER2:342_‐36_FSY_‐CR8 ADCN groups, the red fluorescence was decreased meanwhile the green fluorescence was increased successively, signifying the level of early apoptosis was heightened in sequence. Flow cytometry analysis and quantitative data in Figure 5e,f also revealed the fluorescence intensity of Z_HER2:342_‐36_FSY_‐CR8 ADCN (70.18 ± 4.13%) group was the highest comparing with those of PBS (14.23 ± 0.52%), CR8 (37.84 ± 1.21%), and Z_HER2:342_‐CR8 ADCN (45.09 ± 1.01%) groups.

Apoptosis analysis was further performed using Annexin V‐FITC/PI staining assay. As shown in Figure 5g,h, the highest apoptotic rate was observed in Z_HER2:342_‐36_FSY_‐CR8 ADCN (68.65 ± 1.27%) group compared to those of PBS (2.30 ± 1.23%), CR8 (51.69 ± 2.36%), and the Z_HER2:342_‐CR8 ADCN (63.69 ± 1.07%) groups, which was consistent with the results of JC‐1 test. Furthermore, similar apoptosis results were also observed in ECa‐109 cells (Figure S15, Supporting Information).

Live/Dead assay was conducted by flow cytometry using Calcein‐AM/PI co‐staining test. As shown in Figure 5i,j, the cell death rate induced by Z_HER2:342_‐36_FSY_‐CR8 ADCN was the highest (84.70 ± 0.72%) compared to those by Z_HER2:342_‐CR8 ADCN (71.96 ± 0.90%), CR8 (57.64 ± 1.41%), and PBS (5.30 ± 2.91%). All above results indicated that the degradation of cyclin K induced by Z_HER2:342_‐36_FSY_‐CR8 ADCN effectively triggered apoptosis and resulted in death of cancer cells.

### 2.6 In Vitro and In Vivo Biosafety of Z_HER2:342_‐36_FSY_‐CR8 ADCN

**Figure 6:**
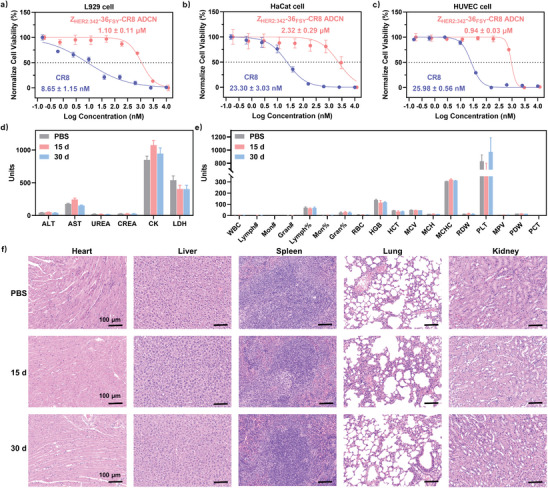
In vitro and in vivo biosafety of Z_HER2:342_‐36_FSY_‐CR8 ADCN. Normalize cell viability of a) L929, b) HaCat, and c) HUVEC cells treated with CR8 and Z_HER2:342_‐36_FSY_‐CR8 ADCN, respectively. d) Blood biochemical and e) hematological parameter analysis of mice on days 15 and 30 after the injection of Z_HER2:342_‐36_FSY_‐CR8 ADCN. Blood biochemical analysis: alanine aminotransferase, ALT; aspartate aminotransferase, AST; blood urea nitrogen, UREA; creatinine, CREA; creatine kinase, CK; lactate dehydrogenase 1, LDH 1. Hematology indicators: white blood cells, WBC; lymphocyte, lymph#; monocyte, Mon#; neutrophils, gran#; red blood cells, RBC; hemoglobin, HGB; hematocrit, HCT; mean corpuscular volume, MCV; mean corpuscular hemoglobin, MCH; mean corpuscular hemoglobin concentration, MCHC; coefficient of variation of erythrocyte distribution width, RDW; platelet, PLT; mean platelet volume, MPV; platelet distribution width, PDW; platelet crit, PCT. f) Hematoxylin‐eosin staining analysis of mice on days 15 and 30 after the injection of Z_HER2:342_‐36_FSY_‐CR8 ADCN, respectively. Scale bar = 100 µm. Data are presented as mean ± SD (*n* = 3).

As a drug candidate, the in vitro and in vivo biosafety of itself is the primary concern for further clinic application. Therefore, we first investigated in vitro biosafety of Z_HER2:342_‐36_FSY_‐CR8 ADCN using the CCK‐8 assay. Three normal cell lines, L929 (mouse fibrosis cells), HaCat (human immortalized epidermal cells), and HUVEC (human umbilical vein endothelial cells), were selected to evaluate the cytotoxicity of Z_HER2:342_‐36_FSY_‐CR8 ADCN to normal cells. As shown in **Figure** **6**a–c, the IC_50_ values of CR8 for L929, HaCat, and HUVEC cells were 8.65 ± 1.15, 23.30 ± 3.03, and 25.98 ± 0.56 nM, respectively. However, the IC_50_ values of Z_HER2:342_‐36_FSY_‐CR8 ADCN for these normal cells were increased three orders of magnitude and reached to 1.10 ± 0.11 µM (L929), 2.32 ± 0.29 µM (HaCat), and 0.94 ± 0.03 µM (HUVEC), respectively. These results indicated that Z_HER2:342_‐36_FSY_‐CR8 ADCN had a higher in vitro biosafety compared to that of CR8, which could be attributed to the HER2‐mediated endocytosis and bio‐triggered release of CR8.

To evaluate in vivo biosafety of Z_HER2:342_‐36_FSY_‐CR8 ADCN, healthy female BALB/c‐Nude mice were randomly divided into three groups (*n* = 3). Two groups of mice were injected with Z_HER2:342_‐36_FSY_‐CR8 ADCN (equiv. dose: 5 mg kg^−1^ of CR8) every 3 d for a total of 5 via the tail vein, which were monitored at 15 and 30 d to assess the short‐term and long‐term toxicity, respectively. PBS‐treated group was used as a control. At the predetermined time, the mice were sacrificed to harvest their blood and major organs for subsequent analysis. As depicted in Figure 6d, no significant difference was observed in the levels of alanine transaminase (ALT), aspartate transaminase (AST), blood urea nitrogen (UREA), creatinine (CREA), creatine kinase (CK), and lactate dehydrogenase 1 (LDH 1) for all treated and control mice groups at 15 and 30 d. This suggested that Z_HER2:342_‐36_FSY_‐CR8 ADCN had a minimal impact on the renal and hepatic functions of mice, which meant its excellent in vivo biosafety.

Furthermore, all hematological parameters, including white cell count and hemoglobin levels, of mice treated with Z_HER2:342_‐36_FSY_‐CR8 ADCN showed no significant differences compared to that of PBS‐treated group (Figure 6e). Additionally, hematoxylin and eosin (H&E) staining of major organs (heart, liver, spleen, lung, and kidney) also revealed no evident pathological damage in all mice groups (Figure 6f). All above results collectively confirmed the in vivo biosafety of Z_HER2:342_‐36_FSY_‐CR8 ADCN.

### 2.7 The Enhanced Tumor Accumulation of Z_HER2:342_‐36_FSY_‐CR8 ADCN

**Figure 7:**
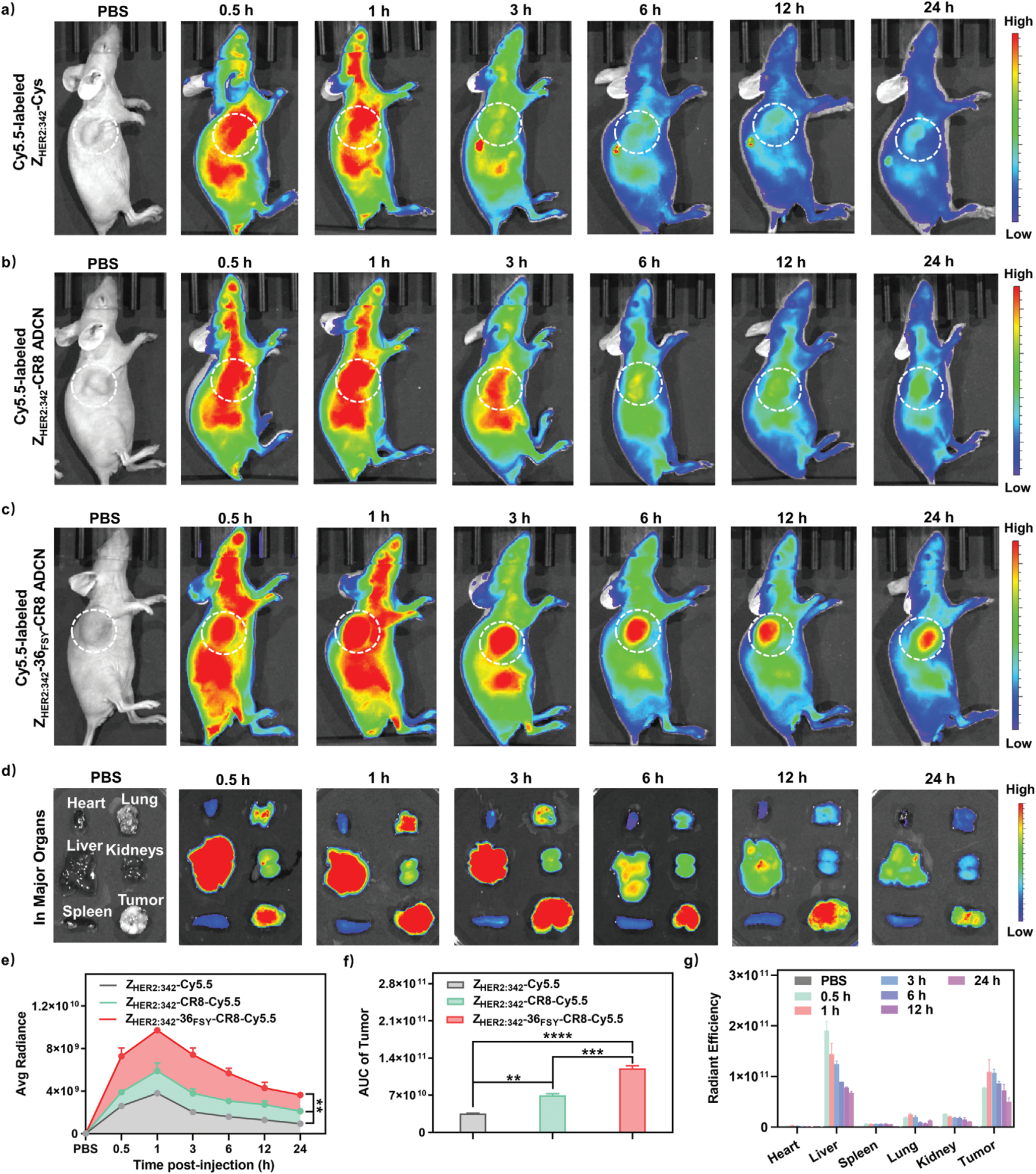
In vivo fluorescence imaging and biodistribution of Z_HER2:342_‐36_FSY_‐CR8 ADCN. In vivo fluorescence imaging in SKOV‐3 tumor‐bearing mice at different time points after the intravenous injection of a) Cy5.5‐labeled Z_HER2:342_‐Cys, b) Z_HER2:342_‐CR8 ADCN, and c) Z_HER2:342_‐36_FSY_‐CR8 ADCN, respectively, at a dose of 1 mg kg^−1^ of Cy5.5. d) Ex vivo imaging of major organs and tumors in SKOV‐3 tumor‐bearing mice at different time points after the intravenous injection of Cy5.5‐labeled Z_HER2:342_‐36_FSY_‐CR8 ADCN. e) Quantitative analysis of avg radiant efficiency in the tumor sites of whole body after 24 h intravenous injection of Cy5.5‐labeled Z_HER2:342_‐Cys, Z_HER2:342_‐CR8 ADCN, and Z_HER2:342_‐36_FSY_‐CR8 ADCN, respectively. f) The integrated area under the curve (AUC) for all treated groups. g) Quantitative analysis of major organs and tumors in SKOV‐3 tumor‐bearing mice at different time points after the intravenous injection of Cy5.5‐labeled Z_HER2:342_‐36_FSY_‐CR8 ADCN. Data are presented as means ± SD (*n* = 3). **p < 0.01, ***p < 0.001, and ****p < 0.0001, *t*‐test.

The exceptional targeting capability of ADCN is beneficial to drug accumulation at tumor sites.^[^ ^11a–c^ ^]^ Here we further investigated the incorporation of covalent‐binding ability into Z_HER2:342_‐36_FSY_‐CR8 ADCN how to affect the in vivo biodistribution of them. As shown in **Figure** **7**a–c, the strong fluorescence signal of Cy5.5 was observed at the tumor site for all treated groups after 1 h post‐injection, which confirmed the excellent tumor targeting ability of Z_HER2:342_ affibody. With the incubation time increasing, the fluorescence signal at the tumor site was rapidly decreased for Z_HER2:342_‐Cys or Z_HER2:342_‐CR8 ADCN treated group, but for Z_HER2:342_‐36_FSY_‐CR8 ADCN treated group the fluorescence signal was declined more slowly, and even maintained at a high level after 24 h post‐injection. We further conducted quantitative analysis of the fluorescence intensity at the tumor sites. As shown in Figure 7e, Z_HER2:342_‐36_FSY_‐CR8 ADCN treated group exhibited a much greater uptake of ADCN at the tumor site within the same time post‐injection compared to that of Z_HER2:342_‐Cys or Z_HER2:342_‐CR8 ADCN treated group. As shown in Figure 7f, the integrated area under the curve (AUC) of Z_HER2:342_‐36_FSY_‐CR8 ADCN treated group was 3.4 or 1.7‐fold higher than that of Z_HER2:342_‐Cys or Z_HER2:342_‐CR8 treated group, respectively. All above data indicated that the incorporation of covalent‐binding capacity into Z_HER2:342_‐36_FSY_‐CR8 ADCN could enhance the tumor uptake and retention time.

Meanwhile, tumors and major organs of Z_HER2:342_‐36_FSY_‐CR8 ADCN treated group were further collected for ex vivo imaging to analyze the in vivo biodistribution of them. As shown in Figure 7d,g, the fluorescence signal of Cy5.5 predominantly accumulated in the liver and tumor sites after post‐injection. Subsequently, the fluorescence intensity in the liver significantly decreased over time, but that in the tumor site remained relatively strong. These results confirmed that the covalent‐binding ability of Z_HER2:342_‐36_FSY_‐CR8 ADCN could give itself longer retention time and effectively accumulate in tumor sites.

The pharmacokinetics of Z_HER2:342_‐36_FSY_‐CR8 ADCN was investigated by analysis of blood samples taken from mice treated with Cy5.5‐labeled Z_HER2:342_‐36_FSY_‐CR8 ADCN. As shown in Figure S16 (Supporting Information), the fluorescence intensity in the bloodstream peaked at 0.5 h (*T*_max_), indicating the rapid absorption of Z_HER2:342_‐36_FSY_‐CR8 ADCN by the mice. Meanwhile, the fluorescence signal could still be detected at a high level of 1246 ± 221.22 a.u. in plasma even after the injection of 24 h, indicating the relatively long blood circulation time of Z_HER2:342_‐36_FSY_‐CR8 ADCN. The calculated half‐life (*T*_1/2_) of Z_HER2:342_‐36_FSY_‐CR8 ADCN in the bloodstream was determined to be 1.37 h (one‐phase decay model), validating its favorable pharmacokinetic profile. These findings further indicated the application potential of Z_HER2:342_‐36_FSY_‐CR8 ADCN in cancer therapy.

### 2.8 In Vivo Anti‐Tumor Activity of Z_HER2:342_‐36_FSY_‐CR8 ADCN

**Figure 8:**
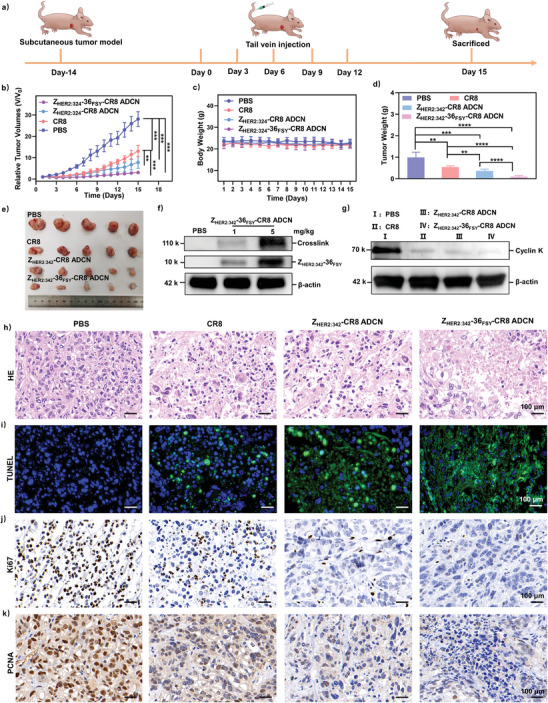
In vivo anti‐tumor activity of Z_HER2:342_‐36_FSY_‐CR8 ADCN. a) Schematic representation of the animal experimental design. b) Relative tumor volume and c) body weight changes of the mice in treatment process. d) Tumor weights and e) gross morphology of the dissected tumors after intravenously injected with PBS, CR8, Z_HER2:342_‐CR8 ADCN, and Z_HER2:342_‐36_FSY_‐CR8 ADCN at the time of sacrifice (*n* = 5). f) Crosslinking of Z_HER2:342_‐36_FSY_‐CR8 ADCN with HER2 in tumor tissues injected with PBS or Z_HER2:342_‐36_FSY_‐CR8 ADCN for 5 h determined by western botting analysis. g) Cyclin K degradability of PBS, CR8, Z_HER2:342_‐CR8 ADCN, and Z_HER2:342_‐36_FSY_‐CR8 ADCN at the time of sacrifice. h) H&E staining, i) TUNEL immunofluorescence staining, and j) Ki67 and k) PCNA immunohistochemistry staining of tumor tissue after intravenously injected with PBS, CR8, Z_HER2:342_‐CR8 ADCN, and Z_HER2:342_‐36_FSY_‐CR8 ADCN at the time of sacrifice (*n* = 3). Scale bar = 100 µm. Data are presented as means ± SD (*n* = 5). **p < 0.01, ***p < 0.001, and ****p < 0.0001, *t*‐test.

Encouraged by the excellent targeting and great accumulation in tumor site of Z_HER2:342_‐36_FSY_‐CR8 ADCN, we subsequently explored the in vivo antitumor activity of them. HER2‐overexpressed SKOV‐3 ovarian carcinoma tumor model mice were established and randomly divided into four groups when tumor volumes reached about 50 mm^3^. Subsequently, mice were intravenously administered with PBS, CR8 (5 mg kg^−1^), Z_HER2:342_‐CR8 ADCN (equiv. dose: 5 mg kg^−1^ of CR8), and Z_HER2:342_‐36_FSY_‐CR8 ADCN (equiv. dose: 5 mg kg^−1^ of CR8) every 3 d for a total of five injections, respectively (**Figure** **8**a).

The tumor volume and body weight of mice were monitored daily throughout the treatment period. As shown in Figure 8b and Figure S17 (Supporting Information), Z_HER2:342_‐36_FSY_‐CR8 ADCN exhibited the significant inhibition of tumor growth after five injections, meanwhile the body weights of mice also showed no discernible changes (Figure 8c), indicating the excellent antitumor efficacy and biosafety of Z_HER2:342_‐36_FSY_‐CR8 ADCN. After the end of treatment, mice were euthanized to collect their tumors for ex vivo weighting and digital imaging (Figure 8d,e). As shown in Figure 8e, the tumors in Z_HER2:342_‐36_FSY_‐CR8 ADCN treated group were significantly smaller than those in other treatment groups. The ex vivo averaged tumor weight of Z_HER2:342_‐36_FSY_‐CR8 ADCN treated group was approximately 0.10 ± 0.04 g, whereas those of PBS, CR8, and Z_HER2:342_‐CR8 ADCN treated groups were 0.99 ± 0.19 g, 0.55 ± 0.05 g, and 0.36 ± 0.07 g, respectively (Figure 8d). The tumor inhibition rate (TIR) of Z_HER2:342_‐36_FSY_‐CR8 ADCN was as high as 90.03 ± 4.29%, which confirmed the remarkable therapeutic efficacy of Z_HER2:342_‐36_FSY_‐CR8 ADCN.

Furthermore, the in vivo covalent targeting capability, and degradation cyclin K ability of Z_HER2:342_‐36_FSY_‐CR8 ADCN were also evaluated by western blotting analysis. As shown in Figure 8f, Z_HER2:342_‐36_FSY_‐CR8 ADCN treated group exhibited the evident dose‐dependent covalent cross‐linking with HER2 in tumor tissues. Moreover, the level of cyclin K in tumor tissues was significantly reduced for CR8, Z_HER2:342_‐CR8 ADCN, or Z_HER2:342_‐36_FSY_‐CR8 ADCN treated group compared to that of PBS‐treated group. Notably, Z_HER2:342_‐36FSY‐CR8 ADCN treated group displayed the most substantial decrease in level of intratumoral cyclin K (Figure 8j). All above results further verified the covalent targeting capacity of Z_HER2:342_‐36_FSY_‐CR8 ADCN, as well as its exceptional ability to degrade cyclin K.

Finally, H&E staining and TUNEL assay were performed to evaluate the apoptotic response of tumor tissues after the end of treatments. As shown in Figure 8h, conspicuous necrosis and fragmentation were observed in Z_HER2:342_‐36_FSY_‐CR8 ADCN treated group, which meant its superior antitumor efficacy in vivo. As shown in Figure 8i, TUNEL staining results also demonstrated a heightened level of cellular apoptosis in Z_HER2:342_‐36_FSY_‐CR8 ADCN treated group compared to that of other treatment groups. In addition, the cell proliferation of tumor tissues was evaluated by Ki‐67 and PCNA staining assay. As shown in Figure 8j,k, Z_HER2:342_‐36_FSY_‐CR8 ADCN treated group demonstrated a significantly lower number of positively stained cells compared to that of other treatment groups, indicating its remarkable ability to suppress tumor cell proliferation. All above results confirmed the excellent therapeutic efficacy of Z_HER2:342_‐36_FSY_‐CR8 ADCN.

## 3 Conclusions

In summary, we successfully designed and prepared a novel affibody‐molecular glue drug conjugate nanoagent through the molecular self‐assembly approach for proximity‐enabled reactive therapeutics. In detail, FSY was incorporated at site 36 of Z_HER2:342_‐Cys via solid‐phase synthesis to produce Z_HER2:342_‐36_FSY_‐Cys, which could specifically target to the proximal histidine at site 490 of HER2. Then a molecular glue drug CR8 was selected to couple with Z_HER2:342_‐36_FSY_‐Cys to form an amphiphilic conjugate. It can self‐assemble into nanoagent (Z_HER2:342_‐36_FSY_‐CR8 ADCN) in PBS. When Z_HER2:342_‐36_FSY_‐CR8 ADCN binds to HER2 target, FSY36 is close to His490 of HER2 to trigger the efficient proximity‐enabled SuFEx reaction, which forms an irreversible covalent bond. This covalent binding mode enhanced the drug accumulation in cancer cells and significantly improved drug retention time in tumor site. Finally, CR8 can be released rapidly by the cleavage of disulfide bonds under the high level of GSH in cancer cells to degrade cyclin K, which further induces the apoptosis of cancer cells. Benefiting from these advantages, Z_HER2:342_‐36_FSY_‐CR8 ADCN exhibits the remarkable therapeutic efficacy for SKOV‐3 ovarian cancer and excellent biosafety. We believe that the development of covalent protein‐drug conjugates will provide a promising avenue for cancer therapy in future.

## 4 Experimental Section

### Synthesis of Z_HER2:342_‐36_FSY_‐CR8 ADCN

Z_HER2:342_‐36_FSY_‐Cys (1 mg, 0.13 µmol) was put into 1.5 mL EP tube and dissolved in 1 mL PBS (pH 7.4). The DMSO solution of CR8‐ss‐Mal (0.17 g, 0.13 mol) was slowly dropped into the Z_HER2:342_‐36_FSY_‐Cys solution. Then, the mixture was continuously stirred for overnight at 25 °C. Finally, a stable self‐ assembled Z_HER2:342_‐36_FSY_‐CR8 ADCN solution was obtained. The resulting solution was dialyzed against PBS using a dialysis bag with a molecular weight cutoff (MWCO) of 3.5 kDa to remove DMSO. The molecular weight of Z_HER2:342_‐36_FSY_‐CR8 was verified by MALDI‐TOF‐MS. Z_HER2:342_‐CR8 ADCN was also synthesized based on the above steps, the following modifications were made: Z_HER2:342_‐Cys was first treated with TCEP (10 µL, 0.5 M) for 3 h, and then dialyzed against PBS using a dialysis bag (MWCO, 3.5 kDa) to eliminate excess TCEP.

### The Critical Micellar Concentration (CMC) Measurement

To ascertain the critical micellar concentration (CMC) value of Z_HER2:342_‐36_FSY_‐CR8 conjugate, Nile Red (NR) was employed as a fluorescent probe. Specifically, an NR acetone solution (2.5 µL, 0.2 mM) was added to 0.5 mL aqueous solutions of Z_HER2:342_‐36_FSY_‐CR8 conjugate at various concentrations. Subsequently, the samples were left at room temperature overnight to completely eliminate acetone. The fluorescent intensity was then measured using a fluorescence spectrophotometer. Each experimental group consisted of three parallel samples for analysis. The CMC value of Z_HER2:342_‐CR8 conjugate was measured according to the above method.

### In Vitro Acid/GSH‐Triggered Payload Release

The in vitro release of CR8 payload from Z_HER2:342_‐36_FSY_‐CR8 ADCN was assessed through the dialysis method under different conditions. In brief, 2 mL of Z_HER2:342_‐36_FSY_‐CR8 ADCN solution (0.125 mg mL^−1^) was placed into dialysis bags with a MWCO of 3.5 kDa. Subsequently, these bags were immersed in 50 mL PBS (pH 7.4) under various conditions. At specified intervals, 1 mL of the buffer solution was withdrawn and replaced with an equal volume of fresh medium. The concentration of released CR8 was determined using a Shimadzu UV1800.

### Crosslinking of Z_HER2:342_‐36_FSY_‐CR8 ADCN with HER2 In Vitro

Purified 0–1 µM solutions of Z_HER2:342_‐Cys, Z_HER2:342_‐36_FSY_‐Cys, or Z_HER2:342_‐36_FSY_‐CR8 ADCN were incubated with 0.5 µM HER2‐ECD in 10 µL of PBS buffer (pH 7.4) at 37 °C for 12 h. Following incubation, 4x Loading Buffer was added to the mixture, and the samples were heated at 95 °C for 10 min. The samples were then separated using SDS‐PAGE and subjected to immunoblotting with a 1:5000 dilution of anti‐His monoclonal antibody.

### Crosslinking of Z_HER2:342_‐36_FSY_‐CR8 ADCN with HER2 on Cells and Tumors

SKOV‐3 cells were cultured in a six‐well plate and allowed to incubate overnight. Subsequently, Z_HER2:342_‐36_FSY_‐Cys (0.5 µM) or Z_HER2:342_‐36_FSY_‐CR8 ADCN (0.5–2 µM) were added to the culture media. After incubating at 37 °C for 5 h, the cells were washed with PBS and then lysed using RIPA buffer containing a protease and phosphatase inhibitor cocktail. The protein samples were quantified using a BCA protein assay kit. Subsequently, the samples were heated for 10 min after the addition of 4x Loading Buffer. These samples were then separated on SDS‐PAGE and subjected to immunoblotting. The anti‐His monoclonal antibody at a 1:5000 dilution was used to probe the target protein. As an internal standard, β‐actin was probed with a 1:5000 dilution of β‐actin antibody.

The tumors were treated with 200 µL of RIPA buffer containing a protease and phosphatase inhibitor cocktail, and then homogenized for lysis. Protein samples were quantified using a BCA protein assay kit. Western blot analysis was subsequently carried out following the same procedures.

### Cellular Uptake

SKOV‐3 and ECa‐109 cells were cultured in 24‐well plates, with pre‐placed cell slides, and incubated overnight at 37 °C. Subsequently, the culture medium containing Cy5.5‐labeled Z_HER2:342_‐CR8 ADCN and Z_HER2:342_‐36_FSY_‐CR8 ADCN at a dose of 10 µg mL^−1^ of Cy5.5, were added and incubated for varying time points (0.5, 1, 2, and 4 h). Following the incubation, the cells were washed with PBS, fixed with a 4% tissue fixative for 20 min, stained with Actin‐Tracker for 30 min, and Hoechst 33342 for 10 min. After sealing, fluorescence imaging was conducted using CLSM. The absorption of Cy5.5‐labeled Z_HER2:342_‐CR8 ADCN and Cy5.5‐labeled Z_HER2:342_‐36_FSY_‐CR8 ADCN at a dose of 10 µg mL^−1^ of Cy5.5 under different conditions were analyzed via flow cytometry.

For the binding specificity assay, SKOV‐3 cells were pre‐incubated with Z_HER2:342_‐Cys (at a dose of 10 µg mL^−1^) for 1 h, followed by treatment with Cy5.5‐labeled Z_HER2:342_‐36_FSY_‐CR8 ADCN (at a dose of 10 µg mL^−1^ of Cy5.5) for an additional 4 h. The cells were then collected and analyzed using flow cytometry.

### The Degradation of Cyclin K In Vitro and In Vivo

To assess the degradation of cyclin K with CR8, Z_HER2:342_‐CR8 ADCN or Z_HER2:342_‐36_FSY_‐CR8 ADCN treatment, a western blot assay was conducted. SKOV‐3 cells were seeded in 6‐well plates and stimulated for 5 h with different formulations (PBS, CR8, Z_HER2:342_‐CR8 ADCN, or Z_HER2:342_‐36_FSY_‐CR8 ADCN). Following stimulation, the cells were washed with PBS three times and then lysed using RIPA buffer. The resulting lysates were subjected to centrifugation at 12000 rpm for 25 min to remove cell debris, and the supernatants were quantified using a BCA kit to determine protein concentrations. Western blotting was carried out using standard procedures.

The tumor tissues were treated with 200 µL of RIPA buffer containing a protease and phosphatase inhibitor cocktail, and then homogenized for lysis. Protein samples were quantified using a BCA protein assay kit. Western blot analysis was subsequently carried out following the same procedures.

### Cytotoxicity Assay

The cytotoxicity of CR8, Z_HER2:342_‐CR8 ADCN and Z_HER2:342_‐36_FSY_‐CR8 ADCN was assessed on a range of cell lines, including HUVEC, HaCat, L929, SKOV‐3, ECa‐109, OVCAR‐3, MDA‐MB‐231, and MDA‐MB‐453 cells, utilizing a CCK8 assay. Cells in the logarithmic growth phase were seeded in a 96‐well plate and cultured overnight. Subsequently, various concentrations of CR8, Z_HER2:342_‐CR8 ADCN or Z_HER2:342_‐36_FSY_‐CR8 ADCN were added to each well, and the cells were incubated for 48 h. Following incubation, the culture medium was removed, and 100 µL of fresh culture medium containing 10 µL of CCK8 solution was added to each well. The plates were then kept at 37 °C for 1–2 h, and the spectrophotometric measurements were taken at 450 nm. Untreated cells served as the negative control, and six replicates were performed for each experimental group.

### Cell Cycle and Apoptosis Assay

SKOV‐3 cells were seeded in 6‐well plates and treated with different formulations (PBS, CR8, Z_HER2:342_‐CR8 ADCN, or Z_HER2:342_‐36_FSY_‐CR8 ADCN) for 48 h at a dose of 200 nM. The cells were then harvested by centrifugation (1000 × *g*, 5 min), and the cell pellets were fixed in 70% ethanol at 4 °C overnight. Subsequently, the cells were suspended in PI/RNase staining buffer (at 37 °C for 30 min). Cell cycle and apoptosis levels were assessed using flow cytometry as per the provided instructions.

### Apoptosis Assay

SKOV‐3 and ECa‐109 cells were seeded in 6‐well plates and treated with different formulations (PBS, CR8, Z_HER2:342_‐CR8 ADCN, or Z_HER2:342_‐36_FSY_‐CR8 ADCN) for 48 h at a dose of 500 nM. Apoptosis was assessed using Annexin V‐FITC and PI staining. The cells were collected, stained with Annexin V‐FITC and PI, and then analyzed using flow cytometry to determine the extent of apoptosis.

### Calcein/PI Cell Viability/Cytotoxicity Assay

SKOV‐3 cells were cultured in 6‐well plates and treated with different formulations (PBS, CR8, Z_HER2:342_‐CR8 ADCN, or Z_HER2:342_‐36_FSY_‐CR8 ADCN) for 48 h at a dose of 500 nM. Cell viability and cytotoxicity were assessed using Calcein/PI staining. The cells were then analyzed via flow cytometry to determine their viability and cytotoxicity.

### In Vivo Safety Evaluation

All animal experiments conducted in this study were ethically approved by the Animal Experimental Ethics Committee of Shanghai Jiao Tong University under Approval No. 20230330‐02.

The heathy female BALB/c‐Nude mice were randomly divided into three groups, each consisting of three mice. Two of these groups were administered Z_HER2:342_‐36_FSY_‐CR8 ADCN at a dose of 5 mg kg^−1^ of CR8 via tail vein injection. These injections were administered five times at 3 d intervals. The remaining group was given an equivalent amount of PBS as a control. After 15 d and 30 d, the mice were euthanized, and their blood and major organs (including the heart, liver, spleen, lung, and kidney) were collected. Blood samples were subjected to routine and biochemical analyses, while the major organs were examined through hematoxylin and eosin (H&E) staining. This allowed for the assessment of potential changes or damage in the major organs.

### In Vivo Fluorescence Imaging, Biodistribution, and Pharmacokinetic Evaluation

To establish a SKOV‐3 ovarian cancer model, SKOV‐3 cells were subcutaneously inoculated into female BALB/c‐Nude mice at 6 weeks of age. When the tumor volume reached a range of 200–500 mm^3^, SKOV‐3 tumor‐bearing mice were intravenously injected with the Cy5.5‐labeled Z_HER2:342_‐Cys, Z_HER2:342_‐CR8 ADCN, or Z_HER2:342_‐36_FSY_‐CR8 ADCN at a dose of 1 mg kg^−1^ of Cy5.5 via the tail vein. Fluorescence images were captured in vivo at different time points, including 0.5, 1, 3, 6, 12, and 24 h after the injection. Subsequently, the mice were euthanized, and both major organs and tumors were collected for fluorescence imaging. The concentration of Cy5.5 in blood serum samples, with equal volumes, was measured to perform pharmacokinetic evaluation using a fluorescence spectrophotometer. Each group had three parallel samples for analysis.

### In Vivo Anti‐Tumor Activity Study

BALB/c‐Nude mice with SKOV‐3 tumor volumes ranging from 50 mm^3^ were divided into four groups, each consisting of 5 mice. These groups included PBS, CR8, Z_HER2:342_‐CR8 ADCN, and Z_HER2:342_‐36_FSY_‐CR8 ADCN. Mice in each group received intravenous injections of 200 µL of CR8, Z_HER2:342_‐CR8 ADCN, or Z_HER2:342_‐36_FSY_‐CR8 ADCN at a dose of 5 mg kg^−1^ of CR8, administered five times with a 3 d interval between each injection. All mice were euthanized on the 15th day, and their tumors were weighted, and extracted for histological analyses, including H&E staining, terminal deoxynucleotidyl transferase‐mediated dUTP nick‐end labeling (TUNEL) staining, and immunohistochemistry of Ki67 and PCNA. Furthermore, body weight, and tumor volumes were recorded on every day throughout the experimental period for subsequent analysis.

### Statistical Analysis

Three independent experiments were carried out for each analysis unless stated otherwise. Continuous variables were expressed as the mean ± standard deviation (SD). The statistical significance between the two groups was measured using the unpaired student's t‐test. Statistical differences are shown as *p < 0.05, **p < 0.01, ***p < 0.001 and ****p < 0.0001.

## Conflict of Interest

The authors declare no conflict of interest.

## Author Contributions

D.Y., X.X., W.H., and W.G. conceived the project. W.G. and X.X. designed, conducted the experiments and wrote the manuscript. X.Y., Q.L., and Y.L. provided relevant experimental technical support and helped with the investigations. W.H., X.X., and D.Y. supervised this study and revised the manuscript. All authors critically revised the article and approved the final manuscript.

## Supporting information



Supporting Information

## Data Availability

The data that support the findings of this study are available from the corresponding author upon reasonable request.
