# Supplementary material for: Covalent Affibody‐Molecular Glue Drug Conjugate Nanoagent for Proximity‐Enabled Reactive Therapeutics
Source: Adv Sci (Weinh). 2025 Jan 17;12(10):2412273. doi: 10.1002/advs.202412273 (PMC11905048; doi:10.1002/advs.202412273)
Supplement: Supplementary file 1 — Supporting Information [file ADVS-12-2412273-s001.pdf]

ADVANCED SCIENCE
Open Access

Supporting Information

for *Adv. Sci.*, DOI 10.1002/advs.202412273

Covalent Affibody-Molecular Glue Drug Conjugate Nanoagent for Proximity-Enabled Reactive Therapeutics

Wenhui Gao, Xiaoyuan Yang, Qingrong Li, Yingchun Liu, Wei Huang, Xuelin Xia* and Deyue Yan**

Supporting Information

Covalent Affibody-Molecular Glue Drug Conjugate Nanoagent for Proximity-Enabled Reactive Therapeutics

*Wenhui Gao, Xiaoyuan Yang, Qingrong Li, Yingchun Liu, Wei Huang, * Xuelin Xia, * and Deyue Yan**

W. Gao, X. Yang, Q. Li, Y. Liu, W. Huang, X. Xia, D. Yan
School of Chemistry and Chemical Engineering, Frontiers Science Center for Transformative Molecules, Shanghai Jiao Tong University
Shanghai 200240, China

W. Huang, D. Yan
XIANGFU Laboratory
Jiaxing Zhejiang 314102, China
E-mail: hw66@sjtu.edu.cn, xuelinxia@sjtu.edu.cn, dyyan@sjtu.edu.cn

Supplementary Methods

Chemicals and Materials

(R)-2-((9-Isopropyl-6-((4-(pyridin-2-yl)benzyl)amino)-9H-purin-2-yl)amino)butan-1-ol (R-CR8) was purchased from Shanghai Bide Pharmatech Co., Ltd. 3, 3'-dithiodipropionic acid, and tris (2-carboxyethyl) phosphine (TCEP) were purchased from Adamas-beta. *N*-(2-Aminoethyl)maleimide trifluoroacetate salt (Mal-NH_2 (TFA)), 2-(7-Azabenzotriazol-1-yl)-*N*,*N*,*N*',*N*'-tetramethyluroniumhexa`fluorophosphate (HATU), *N*,*N*-diisopropylethylamine (DIPEA), and 1-(3-dimethylaminopropyl)-3-ethylcarbodiimide (hydrochloride) (EDCI) were purchased from Sigma-Aldrich. 4-dimethylaminopyridine (DMAP) was obtained from Macklin Biochemical Co., Ltd. Sulfo-cyanine5.5 NHS ester was purchased from Lumiprobe Corporation. All other reagents and solvents were bought from the domestic suppliers and used as received. The cell counting kit-8 (CCK-8) assay, Lyso-Tracker Green, Actin-Tracker Green-488, Mitochondrial membrane potential assay kit with JC-1, crystal violet staining solution, BeyoClick™ EdU cell proliferation kit with Alexa Fluor 594, cell cycle and apoptosis analysis Kit, Calcein/PI cell viability/cytotoxicity assay kit, and Annexin V-FITC/PI apoptosis detection kit were purchased from Beyotime Chemical Reagent Co., Ltd. Hoechst 33342 was purchased from Thermo Fisher Scientific. Extracellular domain (ECD) of HER2 was purchased from Abcam (ab168896). All aqueous solutions were prepared with deionized water (18.2 MΩ·cm, Millipore).

Instrumentation

The proton magnetic resonance (^{1}H-NMR) spectra were recorded by using a Bruker AVANCE III HD 500 spectrometer. Matrix-assisted laser desorption/ionization time of flight mass spectrometry (MALDI-TOF-MS) spectra were recorded by a Bruker Ultraflextreme with autoflex speed TOF/TOF. Circular dichroism (CD) spectra were recorded by CD/J-815 Japan. Tandem mass spectra were recorded by a UltiMate3000 RSLCnano Liquid Chromatography/maXis impact UHR-TOF MS. Laser scanning confocal microscopy (CLSM) images were obtained by a Leica TCS SP8 STED 3X Superresolution multiphoton confocal microscope. Flow cytometry data were recorded by a BD LSRFortessa flow cytometer. Ultraviolet-visible (UV-Vis) spectra were measured by Shimadzu UV1800 and NanoDrop 2000/2000C spectrophotometer. The fluorescence spectra were recorded by using a Tecan & Spark multimode microplate reader. Animal *in vivo* images were recorded by PerkinElmer IVIS Lumina II *in vivo* imaging system.

Cell Lines and Cell Culture

Human umbilical vein endothelial cell line (HUVEC), human immortalized epidermal cell line (HaCat), mouse fibrosis cell line (L929), human ovarian adenocarcinoma cell line (SKOV-3), human esophageal cancer cell line (ECa-109), human ovarian cancer cell line (OVCAR-3), and human breast cancer cell lines, MDA-MB-231, MDA-MB-453, were obtained from the Cell Bank of the Chinese Academy of Sciences (Shanghai, China), and cultured in base medium supplemented with 10% heatinactivated fetal bovine serum (FBS) and 1% Penicillin/Streptomycin. HUVEC, HaCat, L929, and MDA-MB-231 cells were cultured in DMEM medium. ECa-109, and OVCAR-3 cells were cultured in RPMI 1640 medium. SKOV-3 cells were cultured in McCoy's 5A medium. MDA-MB-453 cells were cultured in Leibovitz medium.

Antibodies

β-Actin (CST, 3700), cyclin K (Proteintech, 26933-1-AP), anti-His (Abmart, m20020f), HRP-conjugated Affinipure Goat Anti-Rabbit IgG(H+L) (Proteintech, SA00001-2), and Peroxidase-AffiniPure Goat Anti-Mouse IgG (H+L) (Jackson immunoresearch, 115-035-003).

Synthesis of CR8-ss-COOH

Briefly, 3, 3'-dithiodipropionic acid (219.20 mg, 1.04 mmol), DMAP (8.50 mg, 0.07 mmol), and EDCI (133 mg, 0.70 mmol) were put into a 25 mL vial and dissolved in 10 mL anhydrous DCM under stirred for 0.5 h to activate the carboxylic acid group of 3, 3'-dithiodipropionic acid. The CR8 (30 mg, 0.07 mmol) was subsequently introduced into the vial, and the reaction was protected by nitrogen for overnight. The resulted solution followed by purified by reverse-phase chromatography as white solid.

Synthesis of CR8-ss-Mal

To a solution of CR8-ss (30 mg, 0.05 mmol) in DCM (10 mL), HATU (36.50 mg, 0.10 mmol) and DIPEA (0.5 mL, 0.87 mmol) were added subsequently for 30 min. Then Mal-NH_2 (TFA) (25.4 mg, 0.10 mmol) was subsequently added into the vial, the reaction was stirred at room temperature for overnight followed by purified by reverse-phase chromatography as white solid.

Synthesis of $Z_{HER2:342}$-36_{FSY}-Cys

The preparation of the FSY-modified $Z_{HER2:342}$-Cys is accomplished via solid phase peptide synthesis. The amino acid sequence $Z_{HER2:342}$-36_{FSY}-Cys was provided as follows: HHHHHHVDNKFNKEMRNAYWEIALLPNLNNQQKRAFIRSLYY$_{FSY}$DPSQSANLLAEA KKLNDAQAPKC.

Meanwhile, the fluorosulfate-L-tyrosine HCl salt was synthesized based on the classic SO_2F_2/borax method.[1]

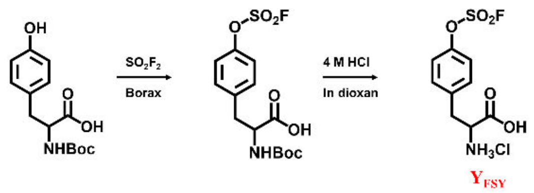

Boc-Tyr-OH (1.00 g, 3.56 mmol) was introduced into a 1 L two-neck round-bottom flask equipped with a magnetic stir bar, along with 210 mL of DCM and 200 mL of a saturated Borax solution. The mixture was vigorously stirred for 6 h. Subsequently, the reaction system was subjected to vacuum until a biphasic solution began to release gas, and this process was repeated three times by refilling it with SO_2F_2. The reaction mixture was vigorously stirred at 25°C overnight. Afterward, DCM was gently removed using a rotary evaporator. Next, 1 M aqueous HCl (100 mL) was added slowly to the reaction mixture while stirring, resulting in the precipitation of a white solid. The mixture was then filtered, and the solid was washed with water for 3 times. The white solid was subjected to vacuum drying at 40°C. This product was utilized directly in the subsequent step without the need for additional purification. Boc-Tyr-OSO_2F (0.4 g, 1.1 mmol) was exposed to 4 M HCl in dioxane (5 mL), and the reaction mixture was stirred overnight. During this period, a white solid precipitated. The solid was then filtered and rinsed with chilled ether, resulting in the isolation of the desired fluorosulfate-L-tyrosine HCl salt as a white solid.

Synthesis of Affibody $Z_{HER2:342}$-Cys

A pET19b-Cys-affibody plasmid was engineered to enable the recombinant expression of $Z_{HER2:342}$-Cys under the control of the IPTG-inducible T7 promoter. These plasmids were separately introduced into E. coli BL21(DE3), and their expression was induced by the addition of 1 mM isopropyl-β-D-thiogalactopyranoside (IPTG) at 16°C overnight. Following induction, the bacteria were subjected to sonication with an ultrasonichomogenizer and subsequently centrifuged at 5000 rpm for 10 min at 4°C. The resulting supernatant was subjected to purification using a Ni-nitrilotriacetyl (Ni-NTA) agarose column. The protein's purity was assessed using 4-20% SDS-PAGE. The purified protein solution was dialyzed against water utilizing a dialysis bag with a molecular weight cutoff (MWCO) of 3.5 kDa and then lyophilized for storage. Finally, the molecular weight of $Z_{HER2:342}$-Cys was confirmed through MALDI-TOF–MS analysis.

Synthesis of Cy5.5-Labeled $Z_{HER2:342}$-36_{FSY}-CR8 ADCN

Sulfo-Cyanine5.5 NHS ester (1 μmol) and $Z_{HER2:342}$-36_{FSY}-CR8 ADCN (0.5 μmol) were combined in a 1.5 mL EP tube and dissolved in 1 mL of D-PBS (pH 7.4). The solution was allowed to stir for 12 h, resulting in a bluish-green solution. To eliminate any unbound Cy5.5,

the solution was dialyzed against PBS using a dialysis bag with a MWCO of 3.5 kDa. The concentration of Cy5.5 was determined using a NanoDrop 2000/2000C spectrophotometer. Cy5.5-labeled $Z_{HER2:342}$-CR8 ADCN, and Cy5.5-labeled $Z_{HER2:342}$-Cys were also synthesized according to the above steps.

Lysosomes Staining

SKOV-3 cells were plated in 4-chamber glass tissue culture slides and left to culture overnight. Subsequently, the culture medium containing Cy5.5-labeled $Z_{HER2:342}$-36_{FSY}-CR8 ADCN (at a dose of 10 $\mu g \cdot mL^{-1}$ of Cy5.5) was added and incubated for 12 h. Following the incubation, the cells were washed with PBS three times and stained with hoechst 33342 (for 10 min) and Lyso-Tracker Green (for 30 min). Finally, the cells were imaged using CLSM, capturing at least three images for analysis.

Colony Formation Assay

SKOV-3 and ECa-109 cells were seeded and cultured in 6-well plates at a density of 500 cells per well for 24 h. Subsequently, the cells were subjected to various treatments, including PBS, CR8, $Z_{HER2:342}$-CR8 ADCN, or $Z_{HER2:342}$-36_{FSY}-CR8 ADCN at a dose of 100 nM. The cells were then cultured for an additional 10 days to permit colony formation. Afterward, the plate was washed with PBS three times. The resulting colonies were fixed with 4% paraformaldehyde for 30 min and stained with 1% crystal violet for 10 min at room temperature. Following another three washes with PBS, the colonies were photographed and quantified. This process was repeated in three separate experiments. The colony formation rate was calculated as the number of colonies in each treatment group divided by the number in the control group, multiplied by 100%.

EdU Assay

The impact of $Z_{HER2:342}$-36_{FSY}-CR8 ADCN on cell proliferation was assessed using the EdU (5-ethynyl-2'-deoxyuridine) assay with the BeyoClick™ EdU Cell Proliferation Kit with Alexa Fluor 594 (Beyotime, C0078S). SKOV-3 cells were seeded in 4-chamber glass tissue culture slides and cultured overnight with different formulations (PBS, CR8, $Z_{HER2:342}$-CR8 ADCN, or $Z_{HER2:342}$-36_{FSY}-CR8 ADCN) for 48 h at a dose of 500 nM. The cells were labeled with EdU (at a dose of 10 μM) for 2 h and then fixed with paraformaldehyde for 15 min. Following fixation, the cells were permeabilized with 0.3% Triton X-100 and then incubated in the dark with the click reaction solution for 30 min. Nucleic acids within the cells were stained with hoechst 33342. Subsequently, the cells were rinsed with PBS containing 3% BSA before examination using CLSM. Furthermore, SKOV-3 cells were cultured in 6-well plates and incubated overnight at 37°C before being exposed to different treatments for 48 h. The EdU assay was

performed according to the provided instructions, and the results were analyzed using flow cytometry.

Cell Scratch Assay

SKOV-3 cells were seeded in a 6-well plate for wound healing assay (n = 3). When the cells reached a confluent state, cells were scraped by a pipette tip. At 24 h after the adding of different treatments (PBS, CR8, $Z_{HER2:342}$-CR8 ADCN, or $Z_{HER2:342}$-36$_{FSY}$-CR8 ADCN) at a dose of 500 nM. The cell images were captured by Leica microscopic imaging system. Then, the wound widths were measured.

Mitochondrial Membrane Potential Assay

SKOV-3 cells were cultured in a 6-well plate and allowed to incubate overnight. Subsequently, 1 mL of culture medium containing different treatments (PBS, CR8, $Z_{HER2:342}$-CR8 ADCN, or $Z_{HER2:342}$-36$_{FSY}$-CR8 ADCN) was added at a dose of 500 nM, and the cells were incubated at 37°C for 48 h. Mitochondrial membrane potential was assessed using the dye JC-1 (Beyotime, C2006). Under normal conditions, JC-1 accumulates in the mitochondria matrix, forming J-aggregates (excitation: 585 nm; emission: 590 nm), which emit red fluorescence. When the mitochondrial membrane potential is disrupted, JC-1 exists as a monomer in the cytosol (excitation: 514 nm; emission: 529 nm), resulting in green fluorescence. The cells were collected, incubated with 1 μg/ml JC-1 at 37°C for 30 min, and then washed with PBS. The samples were subsequently analyzed using CLSM and flow cytometry to assess changes in mitochondrial membrane potential.

Calcein/PI Cell Viability/Cytotoxicity Assay

SKOV-3 cells were cultured in 6-well plates and treated with different formulations (PBS, CR8, $Z_{HER2:342}$-CR8 ADCN, or $Z_{HER2:342}$-36$_{FSY}$-CR8 ADCN) for 48 h at a dose of 500 nM. Cell viability and cytotoxicity were assessed using Calcein/PI staining. The cells were then analyzed via flow cytometry to determine their viability and cytotoxicity.

Supplementary Figures

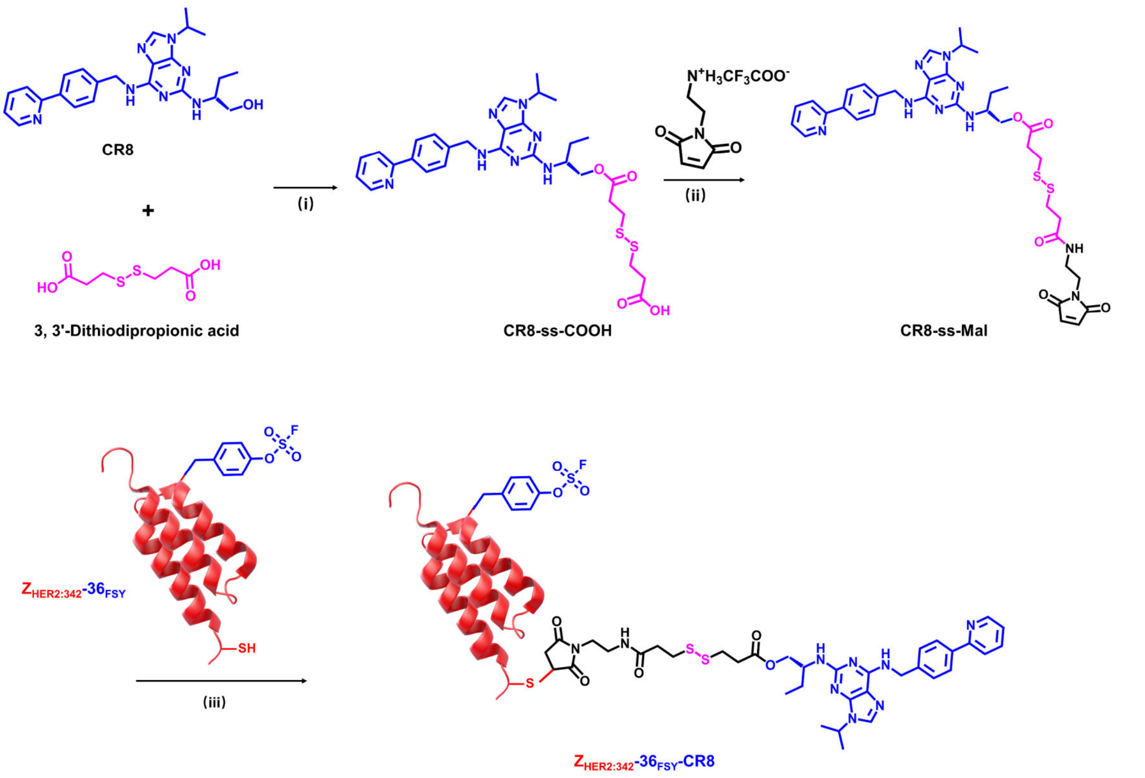

Scheme S1. The synthetic routes of $Z_{HER2:342}$-36_{FSY}-CR8 conjugate. i) DMAP, EDCI, DCM, RT, overnight. ii) Mal-NH_2 (TFA), HATU, DIPEA, DCM, RT, overnight. iii) PBS, RT, overnight. Specific and detailed experimental procedures were provided as described in the above-mentioned methods.

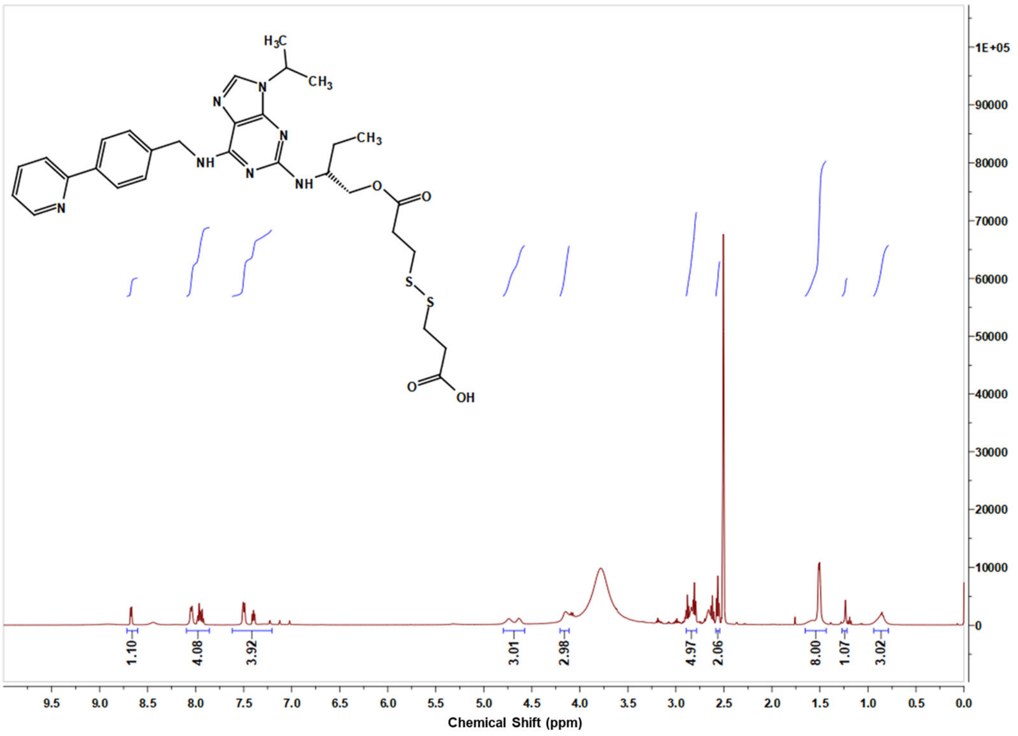

Figure S1. The ^{1}H-NMR spectrum of CR8-ss-COOH.

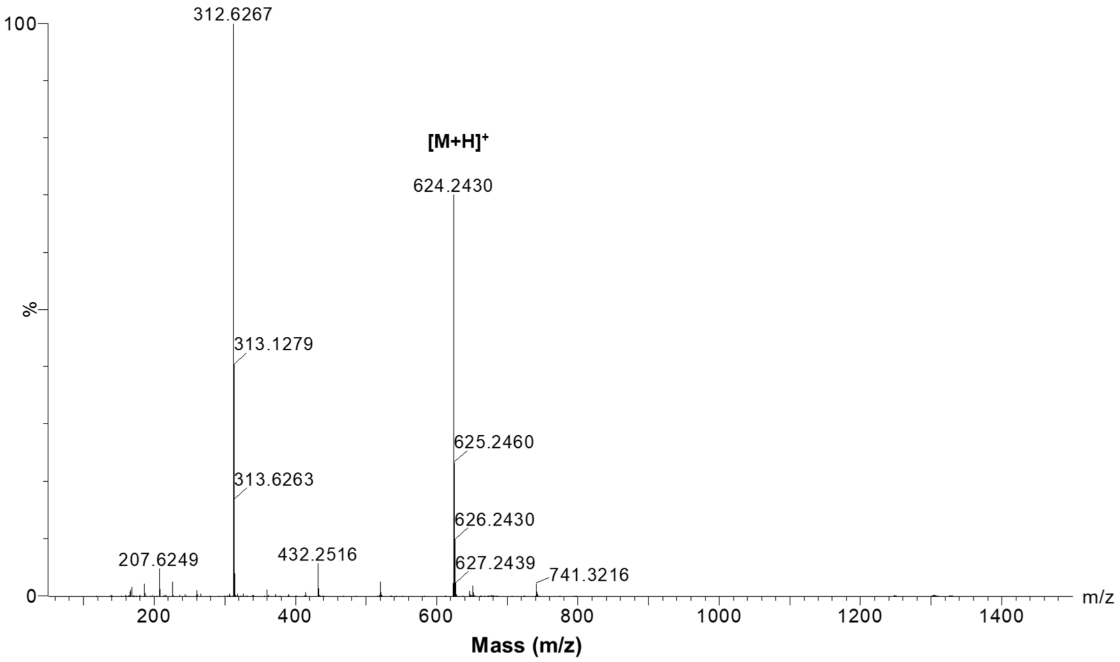

Figure S2. The MS spectrum of CR8-ss-COOH.

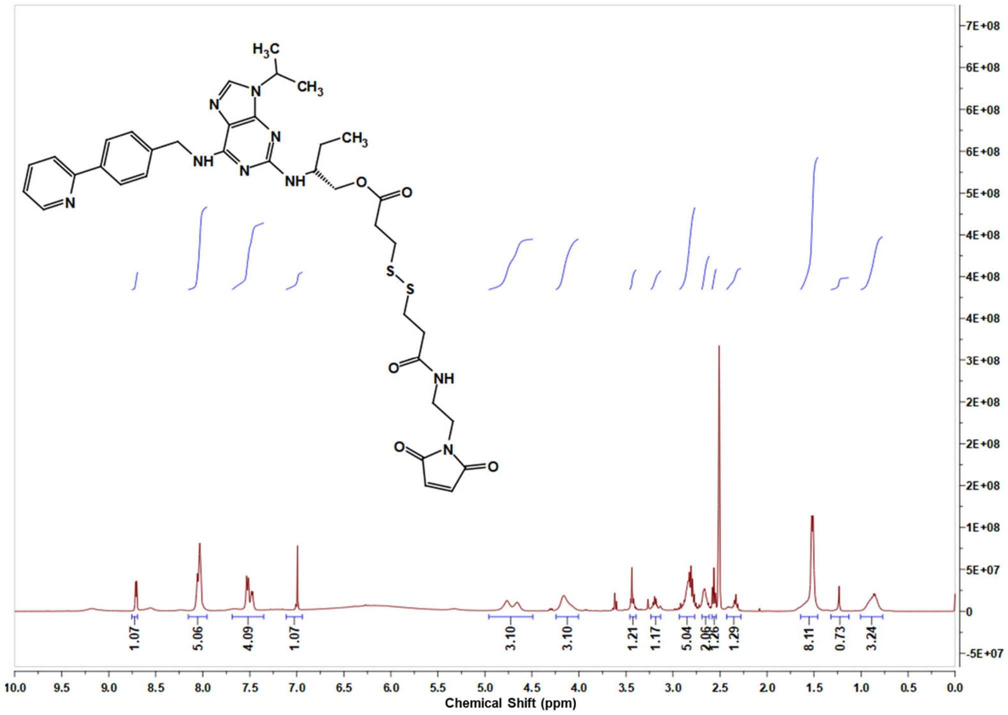

Figure S3. The ^{1}H-NMR spectrum of CR8-ss-Mal.

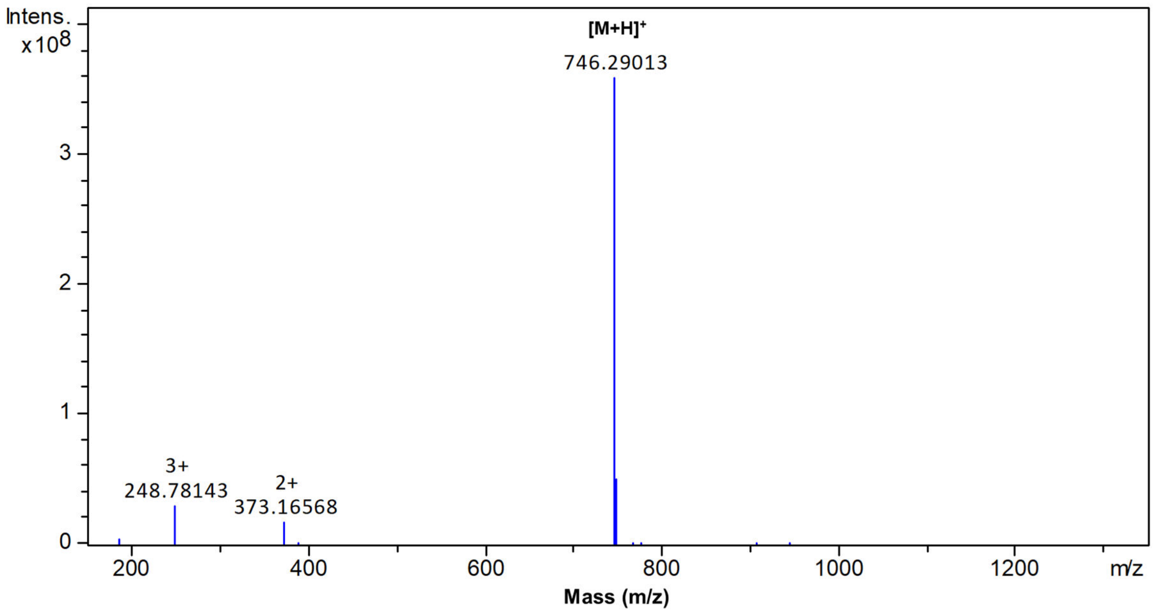

Figure S4. The MS spectrum of CR8-ss-Mal.

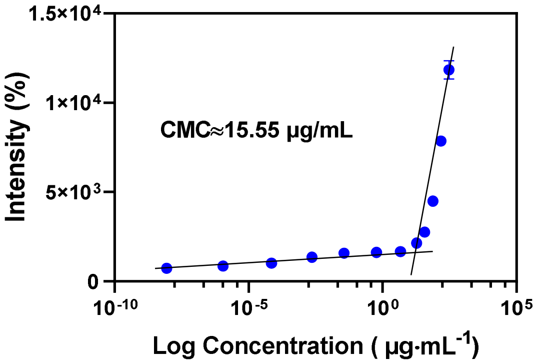

Figure S5. Critical micellar concentration of $Z_{HER2:342}$-36_{FSY}-CR8 conjugate.

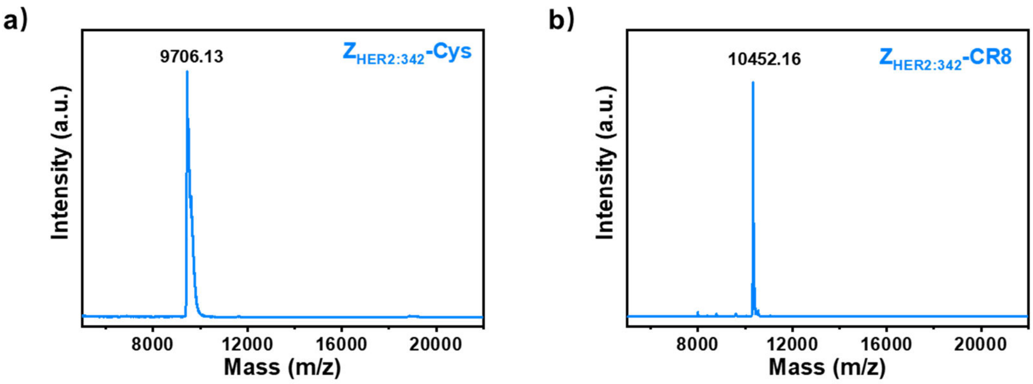

Figure S6. The MALDI-TOF-MS spectra of $Z_{HER2:342}$-Cys (a) and $Z_{HER2:342}$-CR8 conjugate (b).

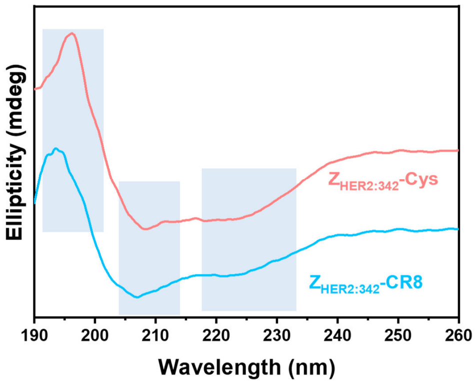

Figure S7. CD spectra of $Z_{HER2:342}$-Cys and $Z_{HER2:342}$-CR8 conjugate.

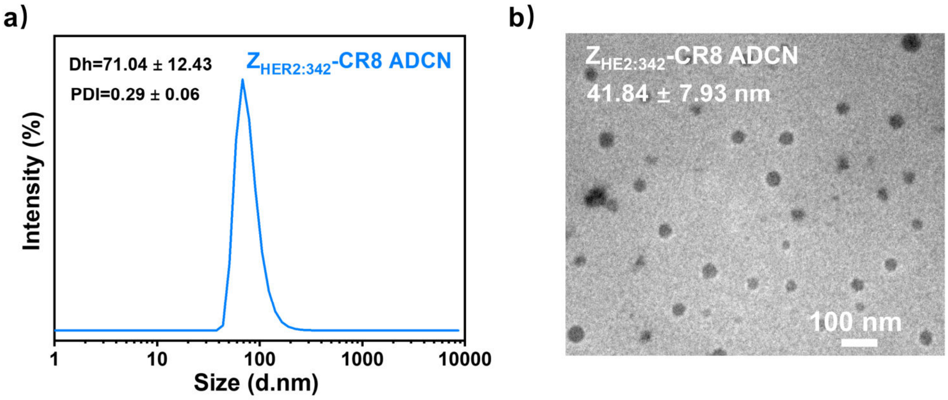

Figure S8. Hydrodynamic diameter of $Z_{HER2:342}$-36$_{FSY}$-CR8 ADCN (a). TEM images of $Z_{HER2:342}$-36$_{FSY}$-CR8 ADCN (b). Scale bar = 100 nm.

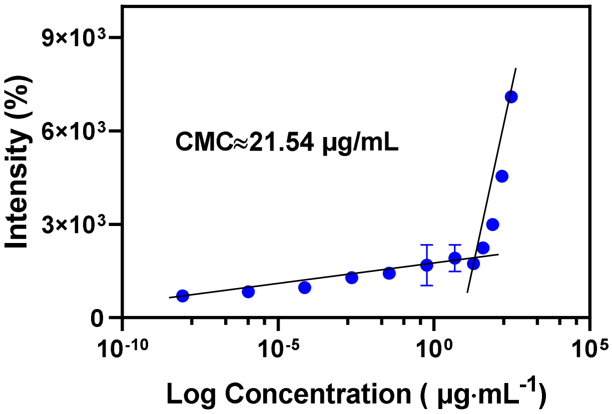

Figure S9. Critical micellar concentration of $Z_{HER2:342}$-CR8 conjugate.

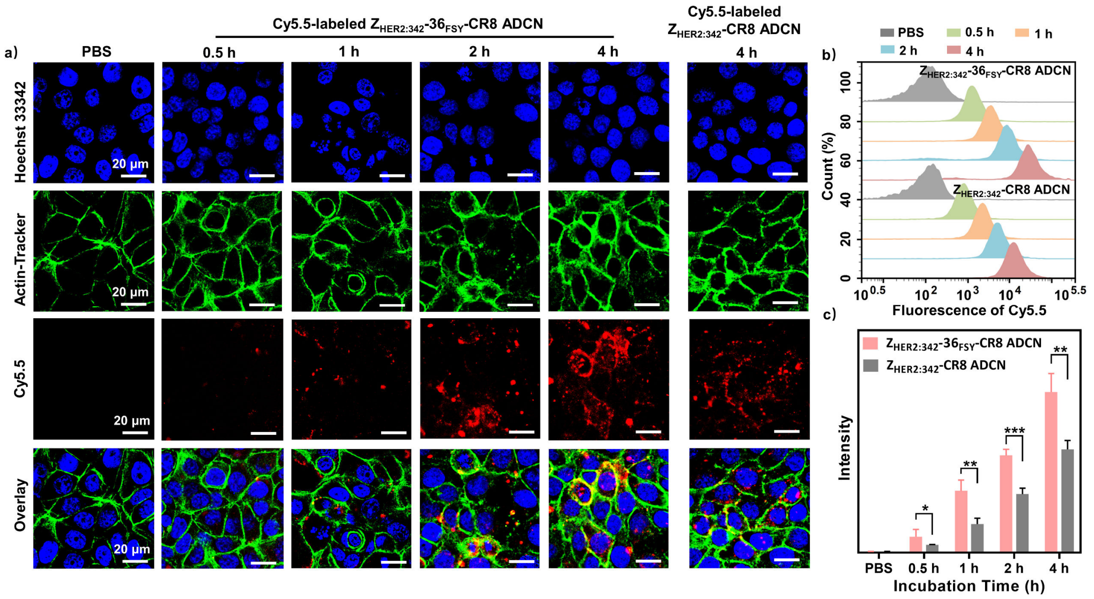

Figure S10. $Z_{HER2:342}$-36_{FSY}-CR8 ADCN increases intracellular uptake. a-c) CLSM images (a), flow cytometry image (b), and flow quantitative data (c) of SKOV-3 cells incubated with Cy5.5-labled $Z_{HER2:342}$-36_{FSY}-CR8 ADCN or Cy5.5-labled $Z_{HER2:342}$-CR8 ADCN (10 μg·mL^{-1} of Cy5.5). Cy5.5 fluorescence (red), actin-tracker (green), and hoechst 33342 (blue). Scale bar = 20 μm. Data are presented as mean ± SD (n = 3). * $p < 0.1$, ** $p < 0.01$, and *** $p < 0.001$. T-test.

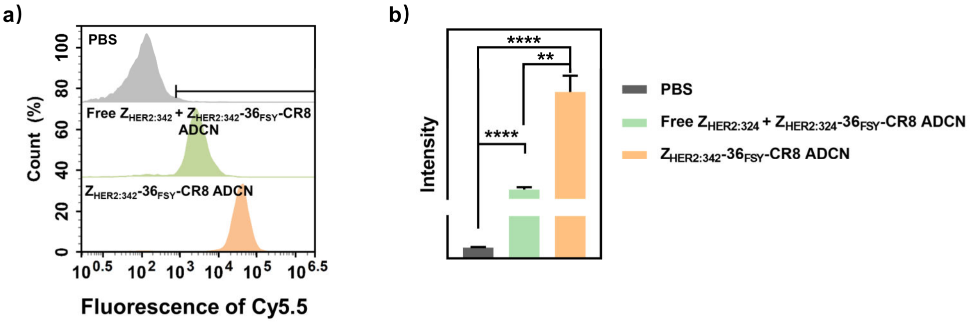

Figure S11. The HER2-mediated endocytosis of $Z_{HER2:342}$-36_{FSY}-CR8 ADCN *in vitro*. a, b) Flow cytometry images (a) and their statistical data (b) of SKOV-3 cells incubated with/without $Z_{HER2:342}$-Cys (10 μg·mL^{-1}) for 1 h and then incubated with Cy5.5-labeled $Z_{HER2:342}$-36_{FSY}-CR8 ADCN (10 μg·mL^{-1} of Cy5.5) for another 4 h. Data are presented as mean ± SD (n = 3). ** $p < 0.01$, and **** $p < 0.0001$, T-test.

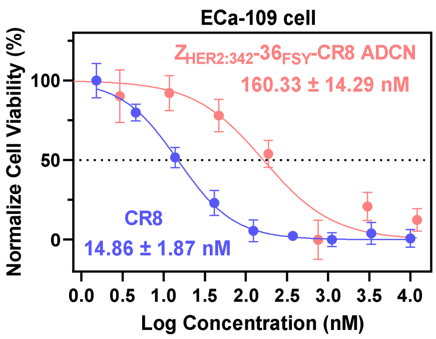

Figure S12. Normalize cell viabilities of ECa-109 cells incubated with free CR8 or $Z_{HER2:342}$-36_{FSY}-CR8 ADCN for 48 h determined by CCK-8 assay.

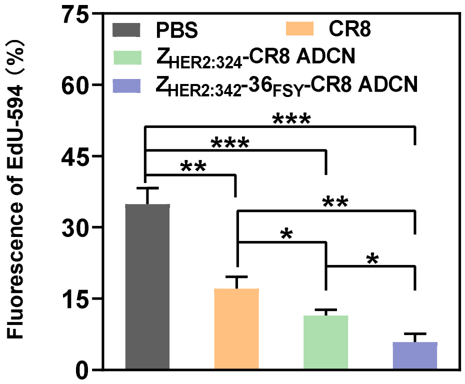

Figure S13. The flow cytometry quantification of EdU-594 assays in SKOV-3 cells treated with PBS, CR8, $Z_{HER2:342}$-CR8 ADCN, or $Z_{HER2:342}$-36_{FSY}-CR8 ADCN, respectively, at a dose of 500 nM. Data are presented as mean ± SD (n = 3). * $p < 0.1$, ** $p < 0.01$, and *** $p < 0.001$, T-test.

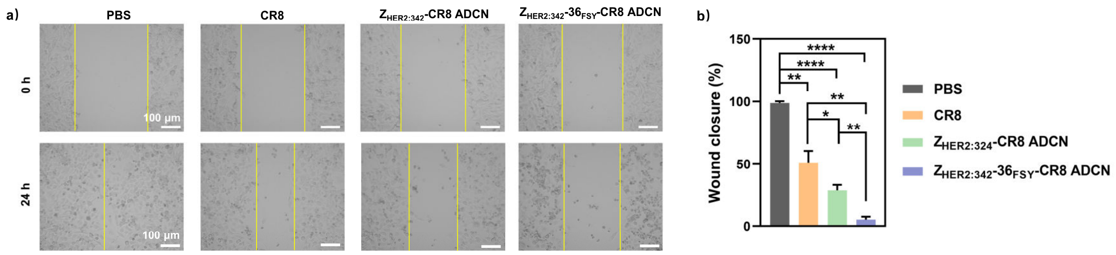

Figure S14. *In vitro* migratory capability of SKOV-3 cells after treated with $Z_{HER2:342}$-36_{FSY}-CR8 ADCN. a, b) Wound healing assay (a) and scratch width (b) of SKOV-3 cells treated with PBS, CR8, $Z_{HER2:342}$-CR8 ADCN, or $Z_{HER2:342}$-36_{FSY}-CR8 ADCN, respectively, at a dose of 500 nM. Data are presented as mean ± SD (n = 3). * $p < 0.1$, ** $p < 0.01$, and **** $p < 0.0001$, T-test.

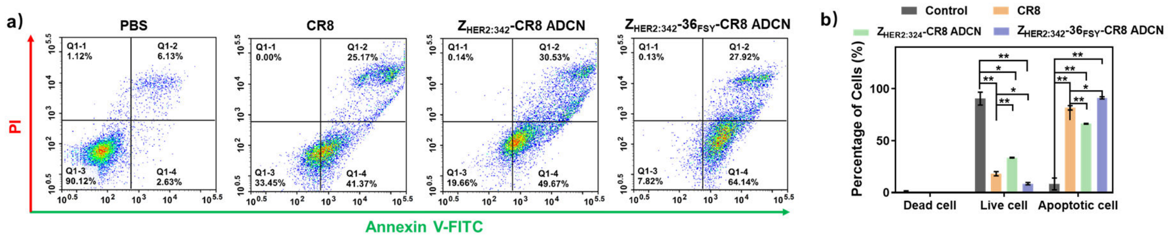

Figure S15. *In vitro* degradation of cyclin K induced cell apoptosis and death. a, b) Flow cytometry images (a) and the corresponding quantitative data (b) of Annexin V-FITC/PI staining assay for ECa-109 cells treated with PBS, CR8, $Z_{HER2:342}$-CR8 ADCN, or $Z_{HER2:342}$-36_{FSY}-CR8 ADCN, respectively, at a dose of 500 nM. Data are presented as mean ± SD (n = 3). * $p < 0.1$, and ** $p < 0.01$, T-test.

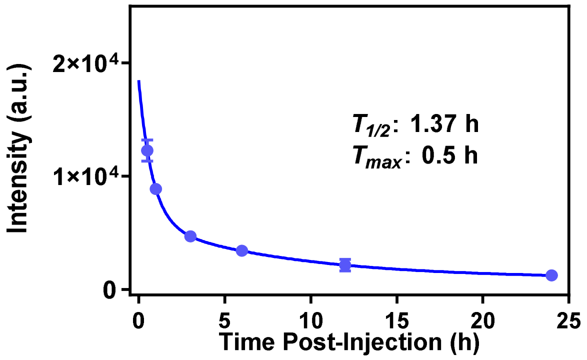

Figure S16. Pharmacokinetics curve of Cy5.5-labeled $Z_{HER2:342}$-36_{FSY}-CR8 ADCN in SKOV-3 tumor-bearing mice.

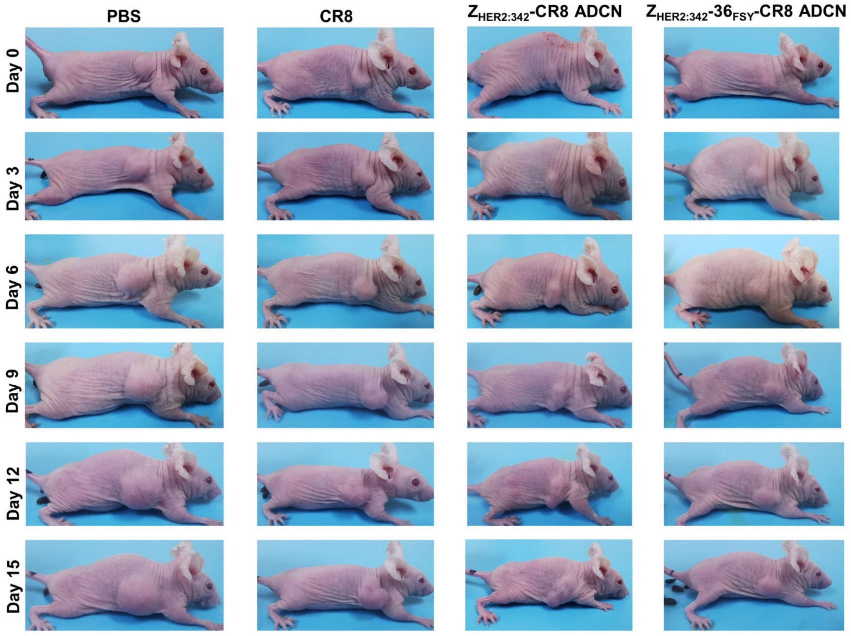

Figure S17. Photograph images changes of mice in treatment process (PBS, CR8, $Z_{HER2:342}$-CR8 ADCN, and $Z_{HER2:342}$-36_{FSY}-CR8 ADCN, CR8 equiv. dose of 5 mg·kg^{-1}).

References

[1] N. Wang, B. Yang, C. Fu, H. Zhu, F. Zheng, T. Kobayashi, J. Liu, S. Li, C. Ma, P. G. Wang, Q. Wang, L. Wang, *J. Am. Chem. Soc.* **2018**, *140*, 4995-4999.
